# Bayesian modelling of population trends in alcohol consumption provides empirically based country estimates for South Africa

**DOI:** 10.1186/s12963-021-00270-3

**Published:** 2021-11-03

**Authors:** Annibale Cois, Richard Matzopoulos, Victoria Pillay-van Wyk, Debbie Bradshaw

**Affiliations:** 1 Division of Health Systems and Public Health, Department of Global Health, Stellenbosch University, Francie van Zijl Drive, Tygerberg, Cape Town, 7505 South Africa; 2 Division of Epidemiology and Biostatistics, School of Public Health and Family Medicine, University of Cape Town, Anzio Road, Observatory, Cape Town, 7925 South Africa; 3 Burden of Disease Research Unit, South African Medical Research Council, Francie van Zijl Drive, Parow Valley, Cape Town, 7505 South Africa; 4 Division of Public Health Medicine, School of Public Health and Family Medicine, University of Cape Town, Anzio Road, Observatory, Cape Town, 7925 South Africa

**Keywords:** Alcohol exposure, Coverage, Bayes, Meta-regression, Trends

## Abstract

**Background:**

Alcohol use has widespread effects on health and contributes to over 200 detrimental conditions. Although the pattern of heavy episodic drinking independently increases the risk for injuries and transmission of some infectious diseases, long-term average consumption is the fundamental predictor of risk for most conditions. Population surveys, which are the main source of data on alcohol exposure, suffer from bias and uncertainty. This article proposes a novel triangulation method to reduce bias by rescaling consumption estimates by sex and age to match country-level consumption from administrative data.

**Methods:**

We used data from 17 population surveys to estimate age- and sex-specific trends in alcohol consumption in the adult population of South Africa between 1998 and 2016. Independently for each survey, we calculated sex- and age-specific estimates of the prevalence of drinkers and the distribution of individuals across consumption categories. We used these aggregated results, together with data on alcohol production, sales and import/export, as inputs of a Bayesian model and generated yearly estimates of the prevalence of drinkers in the population and the parameters that characterise the distribution of the average consumption among drinkers.

**Results:**

Among males, the prevalence of drinkers decreased between 1998 and 2009, from 56.2% (95% CI 53.7%; 58.7%) to 50.6% (49.3%; 52.0%), and increased afterwards to 53.9% (51.5%; 56.2%) in 2016. The average consumption from 52.1 g/day (49.1; 55.6) in 1998 to 42.8 g/day (40.0; 45.7) in 2016. Among females the prevalence of current drinkers rose from 19.0% (17.2%; 20.8%) in 1998 to 20.0% (18.3%; 21.7%) in 2016 while average consumption decreased from 32.7 g/day (30.2; 35.0) to 26.4 g/day (23.8; 28.9).

**Conclusions:**

The methodology provides a viable alternative to current approaches to reconcile survey estimates of individual alcohol consumption patterns with aggregate administrative data. It provides sex- and age-specific estimates of prevalence of drinkers and distribution of average daily consumption among drinkers in populations. Reliance on locally sourced data instead of global and regional trend estimates better reflects local nuances and is adaptable to the inclusion of additional data. This provides a powerful tool to monitor consumption, develop burden of disease estimates and inform and evaluate public health interventions.

## Introduction

Alcohol has widespread, and pervasively harmful, effects on health; its consumption has been identified as a contributing factor for over 200 detrimental conditions, ranging from liver disease and road injuries, to cancers, cardiovascular diseases, psychiatric disorders, tuberculosis and HIV/AIDS [1]. It is estimated that alcohol use in 2016 accounted for 1.6% of the global disease burden in terms of disability adjusted years of life lost among females and 6.0% among males [2].

Despite the solid and growing evidence of the independent role of drinking patterns in determining the health risk associated with alcohol use, the long-term average quantity of alcohol consumed by an individual remains the fundamental predictor of risk [3]. For many conditions, a clear dose-response relationship exists between quantity of alcohol consumed and risk of negative health consequences [4]. In most cases this relationship is monotonic (with higher quantity of alcohol associated with greater risk and no consumption associated with the minimum risk) but there is evidence of J-shaped relationships for some cardiovascular diseases and for diabetes, where low levels of consumption are accompanied by beneficial effects [3, 5, 6]. The evidence is also strong that the dose-response relationship is significantly moderated by sex, and in some cases (e.g. ischaemic heart disease and ischaemic stroke) by age [7–9].

From a public health perspective, the considerations above make it evident that reliable age- and gender-specific population estimates of quantity of alcohol consumed and their temporal trends are key for the correct estimation of the alcohol attributable burden of disease, for designing and evaluating targeted prevention activities and for the rational and efficient planning of treatment services [10]. This information is especially needed in low- and middle-income countries (LMICs), where data on individual consumption are scant but overall sales are often increasing as a result of growing affluence and increased promotional efforts of the alcohol industry [11]. Promotional efforts, moreover, target different demographics that respond differently, resulting on inconsistent trends across age and sex strata.

Producing reliable estimates of alcohol consumption is, however, challenging. As a results, empirically based country estimates are often not available, and reliance is made on global estimation efforts that provide country level estimates, such as those produced by the World Health Organization (WHO) and by the Institute for Health Metrics and Evaluation [1, 2].

There are several reasons that complicated country level estimation. First, survey data on alcohol use—which constitute the main source of information for recovering age- and sex-specific estimates—are almost always based on self-report and suffer from information bias. The bias is usually downward and results in severe underestimation of the actual consumption, with survey data often accounting for less than 50% (but in some cases less than 20%) of the total alcohol sales in a population as recovered from administrative records [12]. It also affects the comparability of the estimates across populations and over time, given the variability of the level of underestimation between surveys, due both to differences in social norms across settings and time which affect the respondents’ level of self-disclosure of their consumption and differences in survey methods and data collection tools, including the set of questions and the reference period for assessing alcohol use (e.g. “last week” vs. “last year”) [13].

Second, survey data are also usually affected by large uncertainties, arising from multiple concurrent factors, including the variable alcohol content of the different drinks; the individual variability of average drink sizes; the fact that the great majority of surveys collect subjects’ responses as intervals (”*one to 3 drinks per week*”) rather than defined quantities.

Third, to be helpful from a public-health perspective, estimation procedures must go beyond reporting the mean of the distribution of alcohol consumption in a population and also provide indications on its (possibly changing) shape. Given the non-linear nature of the dose-response relationship between consumption and risk of disease, the tails of the distribution of alcohol consumption—e.g. the proportions of very low and of very heavy consumers—are of interest as much as the average and only focusing the mean can be severely misleading.

To deal with the limited validity and reliability of survey data, and the consequent ubiquitous underestimation of true consumption, various ‘triangulation’ procedures have been proposed, where administrative data at country level are used to ‘rescale’ survey information on the relative consumption across sex and age categories so that the total consumption across all categories matches the country total recovered from production, sales, import and export statistics. These data are reliably collected in most countries for taxation purposes.

One of those procedures has been developed by Rehm et al. [3, 14], for the calculation of worldwide alcohol consumption trends in the WHO’s *Global status report on alcohol and health* [1]. The basic assumptions underlying this procedure are that: (1) the average daily quantity of alcohol consumed by current drinkers follows a Gamma distribution; (2) in each sex, the standard deviation of the distribution is a linear function of the mean only; (3) the level of underestimation of alcohol consumption in a survey (survey *coverage*) is constant across sex and age categories; (5) the proportion of current drinkers in each age and sex category estimated from survey data reflects the true prevalence in the population.

Assumptions 1 and 2 have significant empirical support, and advantageously substitute unsupported assumptions regarding the characteristics of the distribution, previously used to triangulate survey data with administrative totals, for example in the Comparative Risk Assessment for alcohol within the global burden of disease (GBD) study for the year 2000 [3, 14, 15]. Assumption 3 and 4 are less certain. The empirical evidence regarding how underreporting differs across demographic strata is varied. Studies in general agree that constant coverage is implausible, but the actual level of variability is not consistent across studies [16, 17], and there is some evidence that drinking patterns are stronger predictors of underreporting than demographic factors [18]. The assumption that surveys can provide unbiased estimates of age and sex-specific prevalence of current drinkers is also controversial [19].

In this article we propose a different implementation of the Rehm and Kehoe’s approach where the various steps implied in their methodology are carried out simultaneously in a Bayesian meta-regression framework. We argue that our simultaneous implementation provides an improved quantification of the uncertainty associated with the source data and the estimation procedure itself and a partial relaxation of the assumptions regarding (1) the unbiasedness of the survey prevalence estimates; (2) the relationship between mean and standard deviation of the distribution; and (3) the constancy of the survey coverage. As a further enhancement, in our implementation the censored nature of survey data is taken explicitly into account and the associated uncertainty on individual consumption directly modelled.

In contrast with global models which pool data for various countries and allow global and regional trends to exert a large influence on local estimates [1, 20], our model relies on local data to infer age and sex patterns of alcohol distribution at country level. While global models are advantageous in many circumstances and may produces more reliable estimates in cases where local data is extremely scant and/or of poor quality (and they are the only option in cases where no local data is available), they may also obfuscate local specificities and restrict the use of contextual information and insights [21].

We present here an application of our method to the estimation of age- and sex-specific trends in the prevalence of drinkers and quantity of alcohol consumed by drinkers (in grams of pure ethanol per day) in the adult population of South Africa (15 years and older) between 1998 and 2016.

## Methods

This study adheres to the guidelines for accurate and transparent health estimates reporting (GATHER) recommendations (see Additional file 1: Table A1).

### Data sources

Data on alcohol use at individual level were sourced from 17 surveys conducted in South Africa between 1998 and 2016 on nationally representative samples of the population 15 years and older. Of these, 5 inquired only on presence/absence of current alcohol use, while the remaining 12 collected also information on the quantity consumed by drinkers. A summary measure of the overall risk of bias, the *risk of bias score*, was associated to each survey by using the *Burden of Disease Review Manager* risk assessment tool, developed by the Burden of Disease Unit at the South African Medical Research Council to systematically assess the methodological quality of observational epidemiological studies [22]. The risk of bias score—which takes into account both external (sample representativeness and response rates) and internal validity of the study (appropriateness of definitions and measurement methods)—ranges from 1 to 20, with lower scores indicating higher risk of bias.

Alcohol consumption per capita (APC)—i.e. the total quantity of alcohol consumed by residents in the country divided by the total population 15 years and above—and relative confidence intervals were obtained from the study by Manthey et al. [20] Total APC includes both recorded consumption (derived from official records of alcohol production, import and export and adjusted for tourist consumption) and unrecorded consumption (defined as the quantity of alcohol which escapes official statistic and the usual system of governmental control, such as home or informally produced alcohol, smuggled alcohol, alcohol not intended for human consumption or alcohol obtained through cross-border shopping).

Estimates of the and sex and age structure of the South African population between 1998 and 2016 were provided by the Centre for Actuarial Research (CARe, http://www.care.uct.ac.za/) at the University of Cape Town, and are available in Additional file 2 (Dataset 1).

Additional file 1 (Section 2) includes a complete list of the data sources, details on how they were selected and accessed, and a summary of their characteristics.

### Statistical modelling

We adopted a meta-regression approach to integrate the information on individual consumption patterns extracted from the survey datasets with aggregate data on production, import and export from administrative records.

**Fig. 1:**
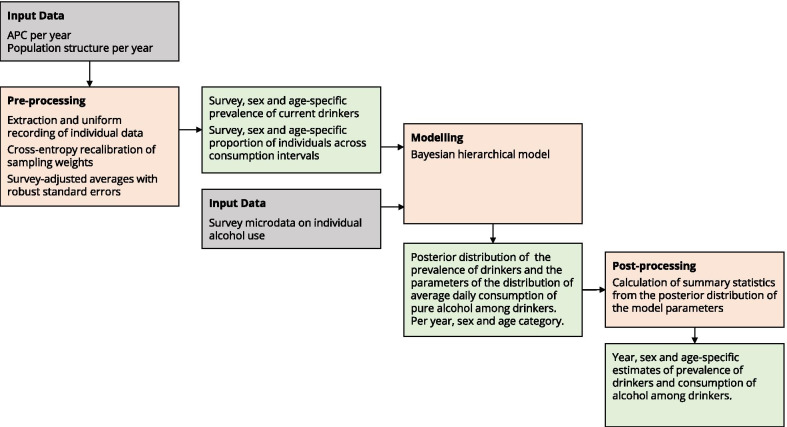
Data analysis method: conceptual overview

We first pre-processed individual level data to calculate, independently for each survey, sex- and age-specific estimates of the prevalence of drinkers and the distribution of individual across consumption categories. We then used these aggregated results, together with data on total APC and population structure, as inputs of a Bayesian model and generated yearly estimates of the prevalence of drinkers in the population and the parameters that characterise the distribution of the average consumption among drinkers, in grams of pure alcohol per day. From the model outputs we calculated the summary measures of interest. Figure 1 provides a conceptual overview of the data analysis method.

#### Pre-processing survey data

From each survey, we extracted data on individual drinking status and estimated the prevalence of current drinkers per sex and 10-years age group (from 15–24 years to 65 years and over).

Of the 12 surveys which collected data on quantity of alcohol consumed, 10 used frequency-quantity questionnaires with discrete sets of responses [23], and two recorded directly the number of drinks consumed in the week preceding the interview. For each participant in the 10 surveys, we calculated an individual range of daily consumption by combining the lower and upper limits of the frequency of alcohol use (number of drinking occasions in a given period of time) and typical quantity (average number of standard drinks per drinking occasion). For each participant in the remaining two surveys, we estimated the individual average consumption by dividing the total number of drinks in the preceding week by seven. We converted the number of standard drinks to grams of alcohol by considering an average content of 12 g of pure alcohol per standard drink, in agreement with the accepted standard for the South African population [12, 24].

We then used these individual consumption data to estimate, separately per survey, sex and age group, the proportion of individuals falling in the different consumption intervals (including the degenerate intervals resulting from the two surveys with direct recording of number of drinks).

In aggregating individual data to estimate the prevalence of drinkers and the distribution across consumption intervals, we took into account the complex sampling scheme of each survey with standard methods (weighted estimators with sandwich-type robust standard errors). To ensure consistency of the sampling weights, we recalibrated the weights with a consistent set of population totals.

With reference to a generic set of *S* surveys and *A* age groups, the output of this process consisted of a set of *np* estimates of prevalence of drinkers ($pp_{s,g,a}$, with standard error $pse_{s,g,a}$) and of *nc* tuples $TC_{s,g,a,k}$ which summarise the distribution of drinkers across consumption intervals:

1 $$\begin{aligned} TC_{s,g,a,k} = \left\{ lc_{s,g,a,k}; uc_{s,g,a,k}; pc_{s,g,a,k}; ne_{s,g,a}\right\} \end{aligned}$$

where:

- $lc_{s,g,a,k}, uc_{s,g,a,k}$ are the bounds of the consumption intervals [g/day];
- $pc_{s,g,a,k}$ is the proportion of subjects belonging to the consumption interval;
- $ne_{s,g,a}$ is the effective sample size;
- $s \in \{1,...S\}$ is the index which identifies the source survey;
- $g \in \{1,2\}$ and $a \in \{1,...A\}$ are the sex and age category indicators;
- $k \in \{1,...K\}$ is the number of different consumption intervals identified across surveys, sexes and age categories.

The effective sample size $ne_{s,g,a}$ is calculated by distributing the total sample across all surveys according to the ‘quality effects weighting’ approach by Doi et al. [25], which allows for integrating in a principled way the information on the precision of the survey estimates (as conveyed by their standard error) with the information of the relative quality of the data sources (as summarised by the risk of bias score).

This relatively complex data structure is justified by the interval-censored nature of the survey data collected with frequency-quantity questionnaires, and avoids introducing the unmodeled error associated with using the middle point of the interval captured by the surveys to represent the actual consumption.

Further details on data pre-processing are reported in Additional file 1.

### Bayesian model

For each year $y \in \{1,...Y\}$ included in the study period and for each sex and age category, the model assumes a Gamma-distributed individual alcohol consumption (Rehm and Kehoe’s Assumption 1), and imposes the constraints that the ratio between standard deviation and mean is approximately constant across sub-populations and time (Assumption 2) and that the sum of the total consumption across age-sex groups multiplied by the survey coverage equals the APC multiplied by the population (basic assumption justifying the triangulation procedure).

As a partial relaxation of Assumption 3, coverage is allowed to vary other than between surveys also across age and sex categories (by the same amount in each survey).

The model also assumes continuity and ‘smoothness’ of the variation across time and age of both the prevalence of drinkers and the mean consumption of alcohol among drinkers.

In statistical terms, the model is expressed by the following likelihood function:

2 $$\begin{aligned}&L = \prod pc_{s,g,a,k} \cdot ne_{s,g,a} \cdot L^{'}_{s,g,a,k} \prod L^{''}_{s,g,a} \end{aligned}$$

3 $$\begin{aligned} & L^{{\prime }} _{{s,g,a,k}} \\ & \quad = \left\{ {\begin{array}{*{20}l} {Gamma(uc_{{s,g,a,k}} |\alpha _{{y,g,a}} ,\beta _{{y,g,a}} /c_{{s,g,a}} )} \hfill & {if\;uc_{ \cdot } = lc_{ \cdot } } \hfill \\ {\int\limits_{{x = lc_{{s,g,a,k}} }}^{{uc_{{s,g,a,k}} }} {Gamma(x|\alpha _{{y,g,a}} ,\beta _{{y,g,a}} /c_{{s,g,a}} )dx} } \hfill & {if\;uc_{ \cdot } > lc_{ \cdot } } \hfill \\ \end{array} } \right. \\ \end{aligned}$$

4 $$\begin{aligned}&L^{''}_{s,g,a} = \mathcal {N}(pp_{s,g,a} \vert p_{y,g,a},pse_{s,g,a}) \end{aligned}$$

where:$\alpha _{y,g,a}$ and $\beta _{y,g,a}$ are the shape and rate parameters of the Gamma distributions which represent the ‘true’ alcohol consumption of drinkers in the specific group (the objective of our estimation) and the two products in Eq. 2 are extended to the *nc* tuples summarising the distribution of consumption and the *np* input prevalence estimates, respectively.

The parameters $c_{s,g,a}$ are a set a positive numbers. Given the scaling property of the Gamma distribution—and assuming that the model is correctly specified—they coincide with the ratio between the observed mean alcohol consumption (from survey data) and the ‘true’ consumption (from the administrative data), i.e. the sex- and age-specific coverage of the survey. We modelled $c_{s,g,a}$ as the product of an overall survey coverage $c^{'}$ times a *coverage deviation* parameter $c^{''}$ which allows variations across age and sex categories:

5 $$\begin{aligned} c_{s,g,a} = c_{s}^{'} \cdot c_{g,a}^{''} \end{aligned}$$

The Gamma parameters $\alpha$ and $\beta$ are expressed in terms of the mean $\mu$ and the standard deviation *sd* of the distribution, with the known formulae:

6 $$\begin{aligned} \alpha _{y,g,a}=\left( \frac{\mu _{y,g,a}}{sd_{y,g,a}}\right) ^2 ;\,\, \beta _{y,g,a}=\alpha _{y,g,a}/\mu _{y,g,a} \end{aligned}$$

The mean $\mu$ is modelled as a smooth function of time and age, separately by gender, with a generalised additive model (GAM) with log link [26].

7 $$\begin{aligned} \log (\mu _{y,g,a}) = \sum _{i=1}^{dc_1} \sum _{j=1}^{dc_2} s^{'}_{g,i,j} \Psi ^{'}_i(year) \Phi ^{'}_j(age) \qquad \forall g \in \{1,2\} \end{aligned}$$

where $\Psi ^{'}_i(year)$ and $\Phi ^{'}_j(age)$ are thin-plate splines bases.

$s^{'}_{g,i,j}$ are real coefficients estimated within the model.

The prevalence of drinkers *p* is similarly modelled with a GAM, were the different link function is chosen to avoid estimated prevalences outside the allowed [0–1] interval:

8 $$\begin{aligned} logit(p_{y,g,a}) = \sum _{i=1}^{dp_1} \sum _{j=1}^{dp_2} s^{''}_{g,i,j} \Psi ^{''}_i(year) \Phi ^{''}_j(age) \qquad \forall g \in \{1,2\} \end{aligned}$$

Rehm and Kehoe’s Assumption 2 is conveyed into the model by imposing the following prior distributions to the shape parameter of the gamma distribution:

9 $$\begin{aligned} \alpha _{y,g,a} = \sim \mathcal {N}(r_g,rs_g) \quad \forall y \in \{1,...Y\},\forall g \in \{1,2\},\forall a \in \{1,...A\} \end{aligned}$$

and the consistency between model estimates and administrative data is formalised by the expression:

10 $$\begin{aligned} \sum _{g=1}^{2}\sum _{a=1}^{A} \mu _{y,g,a} \cdot pop_{y,g,a} \cdot p_{y,g,a} \sim \mathcal {N}((1-w) \cdot apc_y,apcse_y) \qquad \forall y \in \{1,...Y\} \end{aligned}$$

where

$pop_{y,g,a}$ is the proportion of population in each gender-age category in year *y*;

$apc_y$ is the estimated *APC* for year *y* and $apcse_y$ is its standard error;

$w \in [0,1)$ is a numerical coefficient that represents the proportion of APC which is spilled, wasted or stocked and consequently not consumed [13].

Because of Eq. 6, expression 9 imposes an approximately constant ratio between *sd* and $\mu$ across age categories and years.^1^ In light of the evidence provided by Kehoe et al. [14] regarding the variability of this ratio across populations, we set:

11 $$\begin{aligned} r_1 = 1.171^{-2} \approx 0.73\, ;\, rs_1 = 0.028\qquad (males) \\ r_2 = 1.258^{-2} \approx 0.63\, ;\, rs_2 = 0.036\qquad (females) \end{aligned}$$

Expression 10 imposes that, in each year, the weighted sum of the average consumption across all sex-age categories approximately equals the APC in the country corrected for wastage, with a margin of error corresponding to the precision of the available estimates. In agreement with the conservative assumptions used in the WHO Global status report on alcohol and health 2018 [1, p. 399] we set $w = 0.2$. As a sensitivity analysis, we repeated the estimation under the assumption of no wastage ($w=0$).

Finally, we imposed a further constraint to the distribution, which implements the assumption that long-term average consumptions of more than 150 g/day are, if not impossible, extremely unlikely [27, 28]. We implemented this constraint with an informative prior on the 95th percentile of the distribution which assigns an extremely low probability to individual consumptions above 150 g/day. This approach avoids introducing mathematical artifacts consequent to the imposition of ‘hard’ limits to the individual consumption [27], while ensuring that the estimated proportion of individuals with consumption above the limit is negligible for any practical purpose.

Note that we are not assuming completeness of the data structure. In particular, the notation above does not assume that all *S* surveys provide data on prevalence and consumption for each age-sex group, nor that the same consumption intervals are observed within each group.

Additional file 1 provides details on the model structure, on the implementation of the various constraints, and a full list of the prior distributions imposed to the free parameters.

#### Computation and model checking

We implemented and fit the model with *Stan* v. 2.19 [29] and used R v. 3.6 [30] for data manipulation, pre- and post-processing and graphing. We recovered the posterior distribution of the parameters with Stan’s default Non-U-Turn Sampler (NUTS), which is an adaptive version of the Hamiltonian Monte Carlo sampling algorithm [31]. We drew a total of 110,000 samples (10,000 samples from each of 11 parallel chains), discarded the first 60% and used the remaining 44,000 to recover the parameters of interest and the bounds of their 95% credible intervals (CI) as the 50th, 2.5th and 97.5th percentile of the sampled distribution.

We checked the convergence of the sampling algorithm by visually inspecting the trace plots and calculating the Gelman and Rubin potential scale reduction statistic $\hat{R}$ [32], and we calculated the effective sample size (ESS) and the Montecarlo standard error (MCSE) for all parameters as indicators of the reliability of the estimates.

As a posterior predictive checking, we analysed the discrepancies between the predicted and observed distribution of consumption for each survey and we examined the congruence of the distribution of residuals with the modelling assumptions.

## Results

The estimation of the model parameters took approximately 130 hours on a Linux workstation (CPU: Intel^®^ Xeon^®^ E5-1650 v3@3.5GHz; RAM: 16 GB; OS: Ubuntu v. 20.0). Model checking procedures supported the conclusion that the model reached convergence (trace plots assuming the characteristics ‘caterpillar’ shape and $\hat{R}<1.024$ for all parameters), with acceptable values of effective sample size and Montecarlo standard error (ESS $> 539$, MCSE $<5\%$ of the posterior standard deviation for all parameters).

The quantile–quantile plots of the standardised residuals did not suggest major deviations from the assumed normality, both overall and within each survey.

**Fig. 2:**
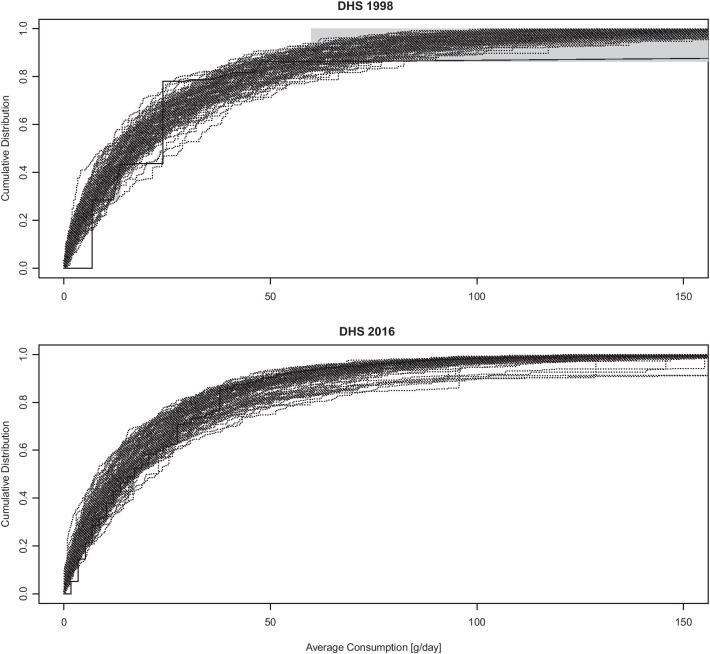
Posterior Predictive Check. Observed versus predicted cumulative distribution of average alcohol consumption among drinkers for the SADHS 1998 and SADHS 2016 surveys. Solid line: observed distribution; Dotted lines: 100 random draws from the posterior distribution. The grey areas represent the zones of uncertainty in the observed distributions for SADHS 1998 due to censoring

The predicted distribution of average consumption among drinkers fit the data reasonably well across surveys. In most cases the observed distribution was comprised within the range of variability of the predictions, with some discrepancies observed for high levels of consumption (above 50–60 g/day) in the three iterations of the SABSSM survey. It must be considered that, because of the censored nature of the data, the ‘observed’ distribution itself is only partially known. As an example, Fig. 2 compares the observed and predicted cumulative distribution of average alcohol consumption among drinkers for the SADHS 1998 and SADHS 2016 surveys. Full results are reported in Additional file 1.

### Prevalence of drinkers and average alcohol consumption among drinkers

**Fig. 3:**
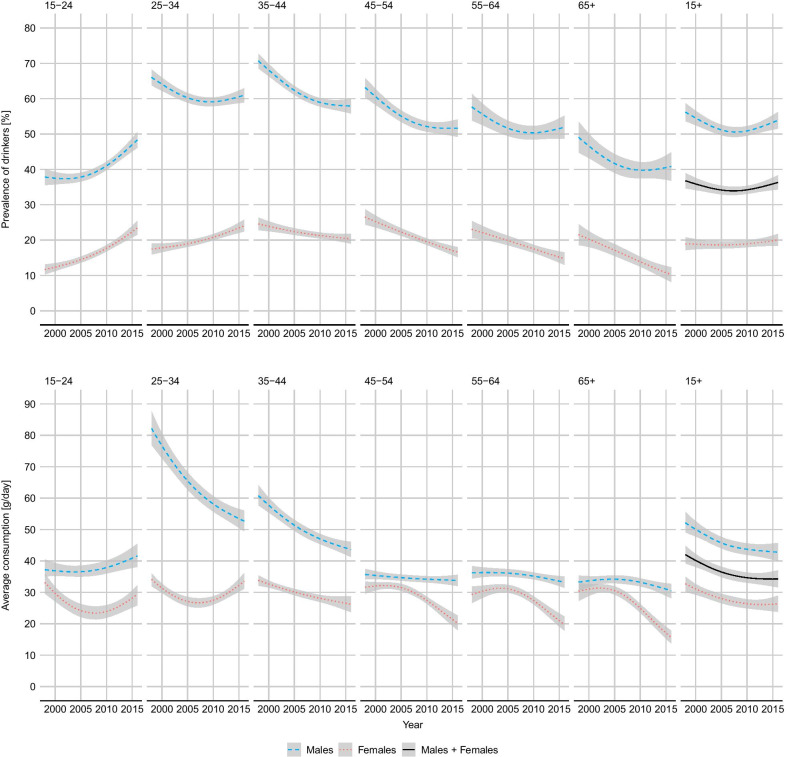
Relative survey coverage per sex and age category. Estimates and 95% credible intervals

Table 1 shows the estimated temporal trends in drinking prevalence and mean consumption among drinkers by sex and for the whole population. Figure 3 depicts age-specific trends (see Additional file 2: Dataset 2 and Dataset 3 for numerical values).

Among males, the prevalence of drinkers rose substantially in the youngest age category (15–24 years), from 37.8% (95% CI 35.6%; 40.1%) in 1998 to 48.3% (46.%; 50.5%) in 2016, and decreased in all other groups. Overall, the prevalence decreased between 1998 and 2009, from 56.2% (95% CI 53.7%; 58.7%) to 50.6% (49.3%; 52.0%), and increased afterwards. In 2016, the estimates prevalence was 53.9% (51.5%; 56.2%).

With the exception of a modest increase among the youngest drinkers, from 37.8 g/day (35.1; 40.4) to 41.6 g/day (38.1; 45.4), the mean consumption decreased in all age groups. The reduction was especially large among the 25–34 years old, whose mean consumption diminished from 82.1 g/day (76.9; 87.5) in 1998 to 52.7 g/day (49.5; 56.0) in 2016 ($-35.8$%). Overall, the mean consumption decreased from 52.1 g/day (49.1; 55.6) to 42.8 g/day (40.0; 45.7).

Among females the increasing trend in prevalence was observed both among the 15–24 years old, from 13.1% (12.2%; 14.0%) to 23.4% (21.5%; 25.4%) and among the 25–34 years old, from 18.0% (16.9%; 19.1%) to 22.9% (21.7%; 24.1%). Overall, the prevalence rose slightly between 1998 and 2016, from 19.0% (17.2%;20.8%) to 20.0% (18.3%; 21.7%).

The overall mean consumption decreased from 32.7 g/day (30.2; 35.0) in 1998 to 26.4 g/day (23.8; 28.9) in 2016. The decreasing trend was driven by the older age groups, while consumption showed a modest increase among subjects under 35 years.

**Fig. 4:**
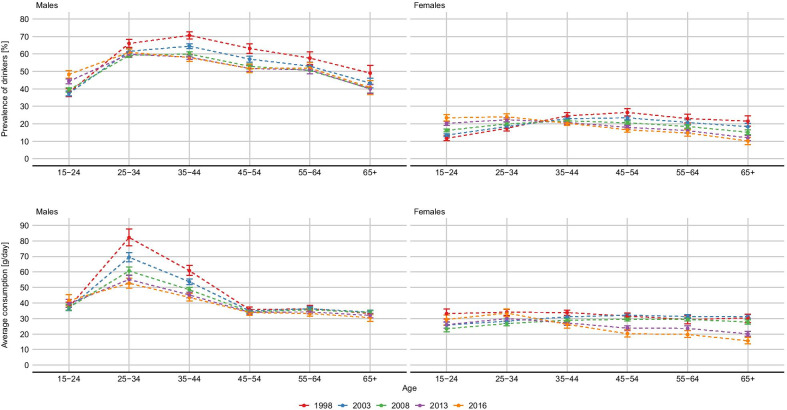
Trends in prevalence of drinkers and mean daily consumption of alcohol among drinkers. South Africa, population 15+, 1998–2016 per sex and age category. Estimates and 95% credible intervals

Figure 4 highlights the changing pattern in the prevalence of drinkers and mean consumption across age categories. The figure suggests a progressive shift of the peak of alcohol consumption towards younger ages. The same trends is observed both among males, and to a greater extent among females.

Across all age categories and years, the shape parameter of the distribution (see Additional file 2: Dataset 2) varied between 0.27 and 1.05 for males and between 0.44 and 0.90 for females, with median 0.64 and 0.66, respectively.

**Table 1:** Estimated prevalence of drinkers and average consumption among drinkers

	Males		Females	
Year	P (%)	C (g/day)	P (%)	C (g/day)
1998	56.2 (53.7; 58.7)	52.1 (49.1; 55.6)	19.0 (17.2; 20.8)	32.7 (30.2; 35.0)
1999	55.4 (53.1; 57.6)	51.0 (48.4; 54.0)	18.9 (17.3; 20.5)	31.9 (29.7; 33.7)
2000	54.5 (52.5; 56.5)	50.1 (47.8; 52.6)	18.8 (17.4; 20.3)	31.1 (29.3; 32.6)
2001	53.7 (51.9; 55.5)	49.1 (47.1; 51.3)	18.8 (17.5; 20.0)	30.4 (28.8; 31.7)
2002	53.0 (51.3; 54.6)	48.2 (46.4; 50.2)	18.7 (17.6; 19.9)	29.7 (28.2; 31.0)
2003	52.3 (50.7; 53.8)	47.2 (45.5; 49.2)	18.7 (17.6; 19.8)	29.1 (27.7; 30.4)
2004	51.7 (50.2; 53.2)	46.5 (44.7; 48.4)	18.6 (17.6; 19.7)	28.5 (27.1; 30.0)
2005	51.2 (49.7; 52.6)	45.8 (44.0; 47.6)	18.6 (17.6; 19.7)	28.0 (26.6; 29.5)
2006	50.8 (49.4; 52.2)	45.1 (43.4; 47.0)	18.6 (17.7; 19.6)	27.6 (26.1; 29.1)
2007	50.6 (49.2; 52.0)	44.7 (42.9; 46.5)	18.7 (17.7; 19.6)	27.2 (25.7; 28.7)
2008	50.6 (49.2; 52.0)	44.3 (42.5; 46.1)	18.7 (17.8; 19.7)	26.9 (25.4; 28.4)
2009	50.7 (49.3; 52.0)	44.0 (42.2; 45.8)	18.8 (17.9; 19.8)	26.6 (25.1; 28.0)
2010	50.9 (49.6; 52.2)	43.7 (42.0; 45.6)	18.9 (18.0; 19.9)	26.4 (24.9; 27.8)
2011	51.2 (49.9; 52.6)	43.5 (41.7; 45.4)	19.1 (18.1; 20.0)	26.2 (24.7; 27.6)
2012	51.7 (50.3; 53.1)	43.3 (41.5; 45.3)	19.2 (18.2; 20.3)	26.1 (24.6; 27.6)
2013	52.2 (50.6; 53.7)	43.2 (41.2; 45.3)	19.4 (18.3; 20.5)	26.1 (24.4; 27.7)
2014	52.7 (51.0; 54.5)	43.1 (40.8; 45.4)	19.6 (18.3; 20.9)	26.1 (24.2; 28.0)
2015	53.3 (51.3; 55.3)	42.9 (40.4; 45.5)	19.8 (18.3; 21.3)	26.2 (24.0; 28.4)
2016	53.9 (51.5; 56.2)	42.8 (40.0; 45.7)	20.0 (18.3; 21.7)	26.4 (23.8; 28.9)

South African adult population [15+], 1998-2016. Per sex

P = Prevalence of drinkers; C = Average consumption among drinkers

95% credible intervals in brackets

### Survey coverage

**Fig. 5:**
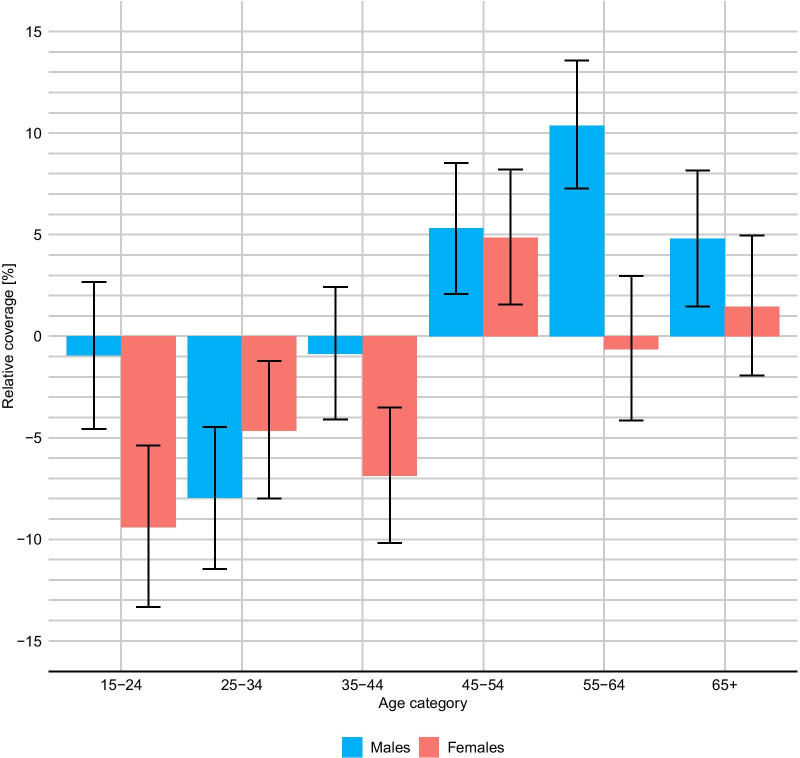
Age and sex patterns in the prevalence of drinkers and mean daily consumption of alcohol among drinkers. South Africa 1998, 2003, 2008, 2013, 2016. Estimates and 95% credible intervals

Survey coverage (Table 2) varied between 27.0% (SABSSM 2008) and 72.7% (SADHS 2016). The age and sex- specific relative coverage (defined as the ratio between the coverage in the group and the overall coverage of the survey) is shown in Fig. 5. Overall, the estimates suggest that females tend to have an higher level of under-reporting than men, and than in both sexes, under-reporting is more severe at younger ages.

**Table 2:** Survey coverage

Survey	Coverage (%)
SADHS 1998	70.3 (66.7; 74.1)
SADHS 2003	54.0 (51.3; 56.9)
NIDS 2008	35.8 (34.1; 37.6)
NIDS 2010	27.9 (26.4; 29.5)
NIDS 2012	35.8 (33.9; 37.8)
NIDS 2014	31.2 (29.4; 33.1)
SABSSM 2005	48.7 (45.6; 52.1)
SABSSM 2008	27.0 (25.6; 28.6)
SABSSM 2012	25.6 (24.2; 27.0)
SANHANES 2012	33.6 (31.9; 35.4)
WHS 2003	52.6 (50.7; 54.5)
SADHS 2016	72.7 (69.1; 76.7)

95% credible intervals in brackets

### Consumption categories

**Fig. 6:**
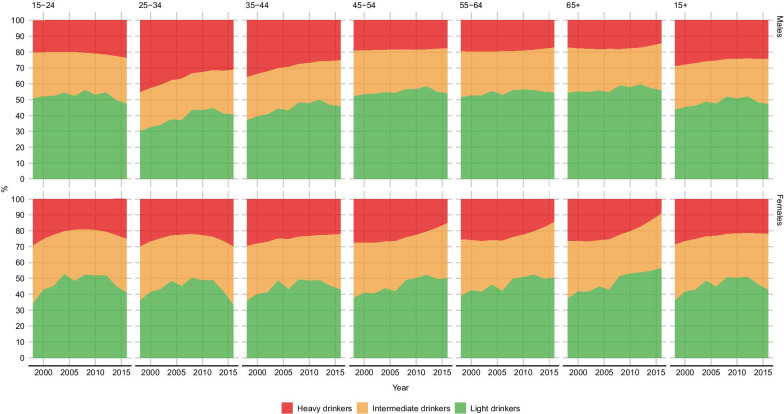
Distribution of drinkers per drinking categories. South Africa, population 15+, 1998–2016.Per sex and age category. *Light drinkers*: average daily consumption < 12/24 g; *Intermediate drinkers*: average daily consumption $\ge$ 12/24 g and < 40/60 g; *Heavy drinkers*: average daily consumption > 40/60 g. The first figure refers to females, the second to males

Figure 6 shows temporal trends in the proportion of light (average daily consumption below 12 g for females and 24 g for males), heavy (average daily consumption above 40 g for females and 60 g for males) and intermediate drinkers.

Overall, the proportion of heavy drinkers decreased steadily among females, from 28.8% (26.2%; 30.9%) in 1998 to 21.9% (19.1%; 24.6%) in 2016. The decrease is mostly due to the consistent downward trends in the oldest age categories, while among the under 35 data suggest that the initial decrease has been reversed in recent years. The proportion of intermediate drinkers has been relatively stable until 2012, but has started increasing afterwards. In 2016, 42.9% (39.9%; 46.0%) of drinkers were classified as light drinkers, and 35.2% (33.6%; 36.8%) as intermediate.

Among males, the overall prevalence of heavy drinkers decreased from 29.0% (27.2%; 31.0%) in 1998 to 24.3% (22.2%; 26.3%) in 2016. Similarly to females, the proportion of intermediate drinkers has been relatively stable until 2012 and started increasing afterwards. In 2016, 47.1% (44.9%; 49.3%) of drinkers were classified as light drinkers, and 28.6% (27.6%; 29.6%) as intermediate. Differently from women, the reduction in heavy drinkers was especially evident in the 25–34 and 35–44 years age groups. Between 1998 and 2016, the proportion of heavy drinkers decreases by 14.2 percentage points in the 25–34 years age group, and by 10.1 percentage points among the 35–44 years old.

During the whole study period, the total volume of alcohol consumed by heavy drinkers was higher that the volume consumed by light and intermediate drinkers together. In 2016, heavy drinkers, with an average of 58.3 g/day, consumed about 58% of the total at country level, compared to 18% of intermediate drinkers (with an average of 33.3 g/day) and 24% of light drinkers (11.5 g/day). Age and sex specific estimates of average consumption and proportion of total consumption by drinking category are available in Additional file 1.

## Discussion

As a response to the substantial evidence suggesting that self-report data on alcohol consumption from nationally representative surveys largely underestimate the true consumption, it is common practice to adjust survey estimates using inflation factors. These are calculated so that the sum of the estimated consumption over the whole population matches the total consumption recovered from administrative data on production, sales, export and import.

Our method adds to various others that have been proposed to deal with the mathematically undetermined problem of calculating the inflation factors and recover an unique set of age- and sex-specific estimates for the prevalence of drinkers and the parameters of the distribution of alcohol consumption among drinkers [3, 33, 34].

The results presented here show that the proposed Bayesian meta-regression approach is feasible and produces plausible results which do not contradict the bulk of evidence regarding the shape of the distribution of average alcohol consumption among drinkers and the variation of consumption patterns with age and between sexes.

The model checking procedure shows that a Gamma distribution is able to adequately recover the distribution of consumption among drinkers in all surveys, age groups and sexes. The range of variation of the estimated shape parameter is consistent with the analyses by Kehoe et al. [14] of 41 datasets across various populations, which observed values ranging from 0.37 to 1.33 for males and from 0.30 to 1.26 for females.

The variations with age of both the prevalence of drinkers and the average consumption among drinkers are also plausible and congruent with the literature. For males, both prevalence and consumption show a rapid increase at young ages, followed by a gradual decrease. Among females, the trend in prevalence is similar (with much lower levels and a peak that happens later in life compared to males). Consumption is, conversely, characterised by a much more modest decrease after the young adulthood peak. Both these trends have been observed previously in other populations (see, for example, Britton et al. [35]).

Absolute levels of coverage for SADHS 2003, SABSSM 2005, 2008 and 2012 and NIDS 2012 are higher than those calculated by Probst at al. [12] Discrepancies are partly explained by the updated APC estimates used in our analyses, by the different prevalence of drinker estimated in our model (in average 18.0% higher across the 5 surveys) and by the different method for calculating the observed average consumption from censored data.^2^ However, the main cause of the differences is the significantly lower estimate of the ‘true’ average daily consumption. Regardless of the difference with previous estimates our data confirms the common finding that population surveys (1) tend to underestimate the true consumption by a large extent and (2) the level of underestimation is highly variable. The fact that SADHS 1998 and SADHS 2016 seem to elicit significantly better information compared to all the other surveys deserves further investigation to identify the underlying reasons.

The model-predicted variations of survey coverage across ages and sexes indicate highest levels of underreporting among young females (15–24 years) and among males 25–34 years, and lowest levels among older males (55–64 years). The lack of data on the actual level of reporting among South African survey respondents precludes a direct verification of our results. However, overall higher level of underreporting at younger rather than older ages may be explained by the fact that younger age groups are those with the highest level of consumption and social desirability bias might explain the tendency to underreport average consumptions perceived as excessive. Specifically for the youngest age group, the fact that the level of underreporting is much higher (almost 10 times) among females than males may be the results of both traditional gender roles (which assigns a positive connotation to alcohol consumption for males, but much less so for females), and also of the negative connotation of alcohol use in pregnancy [36, 37].

Our method—which is based on the *joint* modelling of the prevalence of drinkers and the average consumption among drinkers, subject to the APC constraint—predicts drinking prevalences that are higher compared with estimates based solely on self-report from single sources (such as those by Peltzer and Ramglan [38], Peltzer et al. [39], Vellios and van Walbeek [40]). Our predictions are also generally higher, especially among males, than those from the GBD study [2] and the WHO’s Global Status Reports on Alcohol and Health 2014 and 2018 [1, 41]), which also combine self-report with administrative data but with a different approach. Given that the sum of the drinking prevalence times the average consumption across sexes and age groups is required to match the APC, as an expected consequence the estimated average consumption among drinkers are lower in our study, and, in our opinion, more consistent with realistic expectations regarding the long-term sustainability of the high levels of use for sizeable sectors of the population that are required, for example, to justify average consumptions exceeding 6.7 drinks per day as those reported for 2016 by the WHO.

We believe that our approach has a number of strengths.

First, we took explicitly into account the censored nature of the available data, thus avoiding the introduction of the unmodelled error associated with the reduction of consumption intervals to their middle point.

Second, the formalisation in terms of priors for the model hyperparameters of the assumptions regarding (1) the value of the shape parameter of the Gamma distribution, (2) the APC and (3) the relative coverage across subpopulations, allowed for the inclusion of their uncertainty in the calculations, thus producing, potentially, a better quantification of the error associated with the model predictions. The substitution of the practice of capping the distribution at a fixed (and arbitrary) value with a ‘soft-cap’ also improves the quantification of the error and avoid mathematical artefacts in the treatment of the Gamma distribution.

Third, the joint modelling of prevalence and consumption across multiple years and age groups enabled us to borrow strength across subpopulations, under mild assumption of smoothness of variations over time and age. It also relaxed the assumption of a known prevalence of drinkers and rather leave the relative uncertainty of the prevalence and consumption data to guide the rescaling of the two quantities so that their product is consistent with the APC.

Fourth, the Bayesian approach in the implementation of the model produces as a result the full distribution of model parameters and allows for a post-hoc calculation of various ‘secondary’ statistics (including their credible intervals) that can be of interest more than the mean. An example are the consumption classes reported above, that offer useful insight on the changing drinking habits, not consistent across subpopulations, that underlie the observed variations in the mean consumption.

Fifth, this approach does not require in principle completeness of the data sources (i.e. availability of estimates for all population subgroups at each data point), thus allowing for the integration of data from local surveys.

Various limitations of our study need to be acknowledged.

First, the relative weight attributed to the prevalence and consumption estimates from the individual surveys is based on a combination of the precision of the estimates with a measure of ‘quality’ of the overall survey methodology and realisation, including the the appropriateness of the questionnaire items and the recall period. While the quality effect approach provides a principled way of creating such a combination, the resulting weights are still based on an arbitrary evaluation of the survey quality.

Second, other sources of uncertainty have been neglected, such as those regarding the size of the population within each sex and age group and the proportion of wasted alcohol.

Third, in absence of information for the South African population and the contrasting results in the international literature, we assumed that the differences in coverage across age and sex groups were modest (namely, we modelled relative coverages in a way that made deviations greater than 5% in any direction as extremely unlikely) and, to ensure identifiability of the model, we also assumed constancy over time. Both these hypotheses are arguable.

Finally, further disaggregation of the estimates by geographic, socio-demographic and other stratifiers would increase the relevance of our estimates and allow for finer targeting of public health interventions. Our modelling approach allows, in principle, for the possibility of using further stratification of the population, subject to the availability of reliable (even if sparse) empirical data, and research is underway with this objective.

## Conclusions

Overall, the proposed methodology proved to be a viable alternative to step-by-step approaches to reconcile survey estimates of individual pattern of alcohol consumption with aggregate administrative data and produce meaningful estimates of sex- and age-specific prevalence of drinkers and distribution of average daily consumption among drinkers in populations.

The fact that the model estimates are based on local data without drawing from globally trends or observations from neighbouring countries allows for taking into account local specificities, events and policy interventions that might not be in common with other contexts. This provides a powerful tool for monitoring consumption and inform and evaluate public health interventions. We think that methodological improvements are important to ensure that the alcohol policy debate is informed by accurate prevalence and consumption data. It is certainly not in the interest of public health authorities to have to respond to a range of estimates that have not been properly synthesised. As with smoking, the alcohol industry relies on doubt and confusion to prolong the debate about the feasibility and importance of applying evidence-based interventions and policies.

Further work is needed for improvement of the model, the formal inclusion of additional sources of uncertainty and the validation of the assumptions.

The estimates generated from South Africa confirm previous evidence suggesting that national surveys need to improve their methods for eliciting information on individual alcohol consumption.

## Supplementary information

**Additional file 1.** Additional methods and results.

**Additional file 2.** Additional tables.

## Data Availability

Restrictions imposed by the data user agreement do not allow for including survey microdata as an appendix to this article. Details on how to access the datasets directly from the custodians are included in Additional file 1.

## Abbreviations

APC: Alcohol per capita
CI: Bayesian credible intervals
SADHS: South African Demographic and Health Survey
ESS: Effective sample size
GAM: Generalised additive model
GATHER: Guidelines for accurate and transparent health estimates reporting
GBD: Global burden of disease
MCSE: Montecarlo standard error
NIDS: National income dynamics study
NUTS: Non-U-turn sampler
SABSSM: South African National HIV Prevalence, Incidence, Behaviour and Communication Survey
SANHNANES: South African National Health and Nutrition Examination Survey
WHO: World Health Organisation
WHS: World Health Survey

## Footnotes

1 The best evidence regarding the relationship between mean and standard deviation supports a linear function which includes an intercept for males [13]. In our model, we disregard the intercept and assume a constant ratio between mean and standard deviation, because (1) for realistic values of mean consumption, the influence of the intercept is negligible and (2) in any case, the relationship is only used to assign a reasonable prior to the actual shape coefficient in each age-sex group, but the final value is estimated within the model and it is allowed to vary across groups.

2 In our case the estimation of the average consumption as reported by respondents assumes a Gamma distribution vs. the uniform distribution within each consumption interval implicit in calculation based on the middle point of the interval.
