# Supplementary material for: Bayesian modelling of population trends in alcohol consumption provides empirically based country estimates for South Africa
Source: Popul Health Metr. 2021 Nov 3;19:43. doi: 10.1186/s12963-021-00270-3 (PMC8565040; doi:10.1186/s12963-021-00270-3)
Supplement: Supplementary file 1 — Additional file 1. Additional methods and results. [file 12963_2021_270_MOESM1_ESM.pdf]

Bayesian modelling of population trends in alcohol consumption provides empirically based country estimates for South Africa

Additional File 1
Additional methods and results

Annibale Cois, Richard Matzopoulos, Victoria Pillay-van Wyk, Debbie Bradshaw

1 GATHER Statement **2**

2 Data sources **3**

2.1 Survey data 3

2.2 Alcohol per capita 6

2.3 Population structure 7

2.4 Potential sources of bias 7

3 Additional methods **9**

3.1 Recalibration of sampling weights 9

3.2 Extracting drinking status form survey items 9

3.3 Calculating consumption intervals 11

3.4 Quality effect weighting and effective sample size 12

3.5 Parametrization of the GAM and choice of the number of bases 12

3.6 Soft-capping the distribution 13

3.7 Priors 15

3.8 Stan model and R code 16

4 Additional results **16**

4.1 Model Checking 16

4.1.1 Convergence statistics and posterior distribution of parameters 16

4.1.2 Prevalence/consumption estimates: residuals and posterior predictive check 16

4.2 Sensitivity analysis 19

4.2.1 Increasing spline bases dimension 19

4.2.2 Prevalence and consumption assuming no wasted alcohol 20

4.3 Consumption by drinking category 22

5 References **24**

1 GATHER Statement

This study complies with the Guidelines for Accurate and Transparent Health Estimates Reporting (GATHER) recommendations.[1] The step involved in the analytical procedures and the data sources upon which the estimates reported in this article are based are described as detailed in Table A1 below.

Table A1: GATHER checklist

#	Item	Reference
**Objectives and funding**		
1	Define the indicators, populations, and time periods for which estimates were made.	Main text (*Introduction* and *Methods*).
2	List the funding sources for the work.	Main text (*Declarations*).
**Data Inputs**		
	for all data inputs from multiple sources that are synthesized as part of the study:	
3	Describe how the data were identified and how the data were accessed.	Main text (*Methods*) and Additional File 1 (*Data Sources*).
4	Specify the inclusion and exclusion criteria. Identify all ad-hoc exclusions.	Additional File 1 (*Data Sources*).
5	Provide information on all included data sources and their main characteristics. For each data source used, report reference information or contact name/institution, population represented, data collection method, year(s) of data collection, sex and age range, diagnostic criteria or measurement method, and sample size, as relevant.	Additional File 1 (*Data Sources*).
6	Identify and describe any categories of input data that have potentially important biases (e.g., based on characteristics listed in item 5).	Main text (*Introduction*) and Additional File 1 (*Data Sources*)
	For data inputs that contribute to the analysis but were not synthesized as part of the study:	
7	Describe and give sources for any other data inputs.	Additional File 1 (*Data Sources*)
	For all data inputs:	
8	Provide all data inputs in a file format from which data can be efficiently extracted (e.g., a spreadsheet rather than a PDF), including all relevant meta-data listed in item 5. For any data inputs that cannot be shared because of ethical or legal reasons, such as third-party ownership, provide a contact name or the name of the institution that retains the right to the data.	Additional File 1 (*Data Sources*) and Additional File 2 (*Dataset 1*).
**Data analysis**		
9	Provide a conceptual overview of the data analysis method. A diagram may be helpful.	Main text (*Figure 1*)

10	Provide a detailed description of all steps of the analysis, including mathematical formulae. This description should cover, as relevant, data cleaning, data pre-processing, data adjustments and weighting of data sources, and mathematical or statistical model(s).	Main Text (*Statistical modelling*) and Additional File 1 (*Additional methods*)
11	Describe how candidate models were evaluated and how the final model(s) were selected.	Main Text (*Statistical modelling*) and Additional File 1 (*Additional methods*)
12	Provide the results of an evaluation of model performance, if done, as well as the results of any relevant sensitivity analysis.	Additional File 1 (*Model checking*, *Sensitivity analysis*)
13	Describe methods for calculating uncertainty of the estimates. State which sources of uncertainty were, and were not, accounted for in the uncertainty analysis.	Additional File 1 (*Additional Methods*) and Main Text (*Methdos*, *Discussion*)
14	State how analytic or statistical source code used to generate estimates can be accessed.	Additional File 1 (*Stan model and R code*)
**Results and Discussion**		
15	Provide published estimates in a file format from which data can be efficiently extracted.	Additional File 2 (*Dataset 2*, *Dataset 3*, *Dataset 4*)
16	Report a quantitative measure of the uncertainty of the estimates (e.g. uncertainty intervals).	All estimates are accompanied by 95% credible intervals.
17	Interpret results in light of existing evidence. If updating a previous set of estimates, describe the reasons for changes in estimates.	Main text (*Discussion*)
18	Discuss limitations of the estimates. Include a discussion of any modelling assumptions or data limitations that affect interpretation of the estimates.	Main text (*Discussion*)

2 Data sources

2.1 Survey data

We manually searched the *DataFirst open data repository* at the University of Cape Town (https://www.datafirst.uct.ac.za/dataportal/), the *DHS program data repository* (https://dhsprogram.com/Data/ and the *Human Sciences Research Council data repository* (http://datacuration.hsrc.ac.za/) for population survey data containing participant-level information on current alcohol use. Inclusion criteria for the survey were:

1. Nationally representative sample of the South African population 15 years and older;
2. Data collection conducted after 1994;
3. Including data on alcohol use in the previous year;

Exclusion criteria were:

1. Sub-national target population (in geographic and/or sociodemographic terms);
2. Data collection conducted before 1994;
3. Sampling design and realisation and/or data collection methods not described with sufficient detail to evaluate the level of representativeness and quality.

The search with the specified inclusion and exclusion criteria retrieved the 17 surveys listed in Table A2, with data collection spanning between 1998 and 2016.

Table A2: Data Sources

Survey	Edition	Acronym	Collection*	Sample size†	Quant‡	Reference
World Health Survey	2003	WHS 2003	2003	351	YES	[2]
South African Demographic and Health Survey	1998	SADHS 1998	1998	13 786	YES	[3]
	2003	SADHS 2003	2003	8 089	YES	[4]
	2016	SADHS 2016	2016	10 336	YES	[5]
National Income Dynamics Study	2008	NIDS 2008	2008	15 502	YES	[6]
	2010-11	NIDS 2010	2010-11	16 636	YES	[7]
	2012	NIDS 2012	2012	18 651	YES	[8]
	2014-15	NIDS 2014	2014-15	22 723	YES	[9]
South African Social Attitudes Survey	2003	SASAS 2003	2003	4 955	NO	[10]
	2004	SASAS 2004	2004	5 596	NO	[11]
	2010	SASAS 2010	2010	3 056	NO	[12]
	2014	SASAS 2014	2014	3 073	NO	[13]
South African National Health and Nutrition examination Survey	2012	SANHANES 2012	2012	4 980	YES	[14]
South African National HIV Prevalence, Incidence, Behaviour and Communication Survey	2002	SABSSM 2002	2002	7 060	NO	[15]
	2005	SABSSM 2005	2005	16 116	YES	[16]
	2008	SABSSM 2008	2008	13 097	YES	[17]
	2012	SABSSM 2012	2012	26 316	YES	[18]

* year(s) of data collection; †number of adult individuals (15 years and older) with non-missing data on alcohol consumption; ‡presence (YES) or absence (NO) of data on quantity of alcohol consumed

Further 15 surveys – subsequent iterations of the All Media Products Survey (AMPS) carried out annually by the South African Audience Research Foundation (SAARF) to collect data on ownership or usage of certain products and services – were also identified in the repositories, but excluded from the analysis for lack of information on sampling design and realisation. A further nationally representative survey – the South African Stress and Health (SASH) Study[19] – was suggested for inclusion by a member of the expert group which consult the Burden of Disease Research Unit on substance abuse related matters. We were, however, unable to access the microdata, which are not in the public domain.

Of the 17 surveys in Table A2, the WHS 2003 and NIDS datasets are publicly available from *DataFirst* at the University of Cape Town (https://www.datafirst.uct.ac.za/dataportal/). The

DHS 1998 and DHS 2016 datasets are also in the public domain and available for download from the *DHS program data repository* (https://dhsprogram.com/Data/). The SABSSM, SASAS and SANHANES dataset are available for download from the *Human Sciences Research Council data repository* (http://datacuration.hsrc.ac.za/), subject to approval by the curator. The DHS 2003 dataset is not on the public domain, but a copy can be requested to the *South African National Department of Health* through the *National Health Research Database* (https://nhrd.hst.org.za/).

In all surveys, the target population was the South African population resident in private households, workers' hostels, convents and monasteries. The data frame excluded other collective living quarters, such as student hostels, old age homes, hospitals, prisons and military barracks. In all cases the target population included subjects 15 years old and above, and in some cases also younger individuals (of no interest for this study and excluded from the datasets). With the exception of the NIDS, all surveys are of cross-sectional design. The NIDS datasets, on the contrary, represent repeated cross-section of members of a panel study initiated in 2008 and still ongoing. The cross-sections considered here include both members of the original panel and additional individuals co-habiting with the panel members at the moment of the data collection.

Data on current alcohol use and, when available, on quantity consumed were self-reported by participants in response to a set of direct questions. The exact set of questions varies across surveys. In particular:

1. the different iterations of the SASAS and the 2002 iteration of the SABSSM only included binary questions enquiring on the current and past use of alcohol, while the remaining 12 surveys also included question on frequency of drinking and quantity of alcohol consumed in a typical occasion (NIDS, SABSSM 2005 and subsequent, SANHNES, SADHS 1998 and 2003) or on the average consumption in the previous week (SADHS 2016 and WHS 2003);

2. the reference period to distinguish between *former* and *current* drinkers and to recall alcohol consumption varied across surveys, as per Table A3 below:

Table A3: Recall period

Survey	Recall period: Current Drinking†	Recall period: Consumption‡
WHS 2003	Unspecified	7 days
SADHS 1998	Unspecified	Unspecified
SADHS 2003	12 months	12 months
SADHS 2016	12 months	7 days
NIDS 2008-2014	Unspecified	Unspecified
SASAS 2003, 2004	Unspecified	-
SASAS 2010, 2012	12 months	-
SANHANES 2012	12 months	Unspecified
SABSSM 2002	Unspecified	-
SABSSM 2005-2012	12 months	Unspecified

†= having had one or more drinks in this period defines a *current drinker*; ‡= recall period to calculate the *frequency of drinking/average consumption.*

For all sources, details on the sampling strategy and realisation (including response rates at the different levels of clustering), survey questionnaire and other metadata are publicly available from the repositories above and/or the references in Table A2.

2.2 Alcohol per capita

Table A4 shows yearly estimates of recorded, unrecorded and total alcohol consumption per capita (APC) between 1998 and 2016 obtained from the study by Manthey et Al.[20][*] and converted from litres per year to grams per day by using the conversion equation:[21]

$$1\,l\;pure\;alcohol \equiv 793\,g \tag{A1}$$

The 95% confidence intervals for total APC in the table are obtained by approximating the standard error (se) with the relative uncertainty related to recorded/unrecorded APC:[20, p. 4]

$$se_{APC} = \frac{0.1 \cdot APC_{recorded} + 0.5 \cdot APC_{unrecorded}}{\sqrt{100}} \tag{A2}$$

and then calculating the bounds of the intervals with the normal approximation.

Table A4: Estimated recorded, unrecorded and total alcohol consumption per capita in South Africa between 1998 and 2016 [g/day per population 15+]

	Recorded	Unrecorded	Total		
Year	APC	APC	APC	lb	ub
1998	23.2	4.6	27.8	26.9	28.7
1999	21.8	5	26.8	25.9	27.7
2000	20.4	5.3	25.7	24.8	26.6
2001	18.7	5.1	23.8	22.9	24.6
2002	18.2	5.2	23.4	22.5	24.3
2003	17.8	5.3	23.0	22.2	23.9
2004	18.2	5.6	23.8	22.9	24.7
2005	18.6	6.0	24.5	23.6	25.5
2006	19.1	6.4	25.6	24.6	26.6
2007	18.9	6.6	25.5	24.5	26.5
2008	18.4	6.6	25.0	24.0	26.0
2009	17.2	6.5	23.7	22.7	24.7
2010	16.3	6.4	22.6	21.7	23.6
2011	15.7	5.9	21.6	20.7	22.5
2012	15.7	5.6	21.2	20.4	22.1
2013	15.6	5.3	20.8	20.0	21.7
2014	15.5	5.0	20.4	19.7	21.2
2015	15.4	4.7	20.1	19.4	20.9
2016	15.3	4.7	20.0	19.3	20.8

APC = alcohol per capita; lb, ub = lower, upper bound of the 95% confidence interval.

[*] The published article only reports a subset of the estimates used for this analysis. The remaining figures were obtained directly from the Authors.

2.3 Population structure

Estimates on the distribution of the South African adult population (15+) across sex and 10-years age groups for each year between 1998 and 2013 were obtained from the Centre for Actuarial Research (CARe, http://www.care.uct.ac.za/) at the University of Cape Town. Distributions for 2014, 2015 and 2016 were approximated by linear extrapolation of the last two years (see Dataset 1, Additional File 2).

2.4 Potential sources of bias

The general limitations of survey data highlighted in the main text (likely underreporting of consumption, uncertainty of alcohol content of the various types of drinks, categorisation of responses, incoherence or lack of precise definition of reporting periods) apply to the data sources considered for this study. Of particular relevance:

1. Current use and quantity consumed are all self-reported and thus subject to a potentially large degree of bias.
   Probst and colleagues[22] investigated the level of underreporting for some of these surveys (SADHS 2003, SABSSM 2005, 2008 and 2012, NIDS 2012) and found that it varies between -84% to -72% (with reference to *recorded* alcohol consumption);

2. as shown in Table A3, the reference period for distinguish former from current drinkers is not completely consistent across surveys.
   While the relationship between recall period and bias is a complex one and moderated by drinking patterns, longer recall periods are generally associated with higher levels of underreporting.[23, 24]

3. the categories used to quantify drinking frequency and typical quantity per drinking occasion are not identical across surveys.

Table A5 provides a summary measure of the risk of bias associated with alcohol related questions in the 17 data sources, as quantified by the *Burden of Disease Review Manager* BODRevMAN risk assessment tool.[25] The risk of bias scores shown in the table range from 1 to 20, with lower scores indicating higher risk of bias (1 to 6: High risk; 7 to 12: Moderate risk; 13 to 20: Low risk).

Table A5: Risk of bias score associated with alcohol-related items, per survey

Survey	Edition	Risk of bias score	Category of risk
World Health Survey	2003	13	Low
South African Demographic and Health Survey	1998	13	Low
	2003	15	Low
	2016	15	Low
National Income Dynamics Study	2008	13	Low
	2010-11	10	Moderate
	2012	10	Moderate
	2014-15	10	Moderate
South African Social Attitudes Survey	2003	10	Moderate
	2004	10	Moderate
	2010	10	Moderate
	2014	10	Moderate
South African National Health and Nutrition examination Survey	2012	14	Low
South African National HIV Prevalence, Incidence, Behaviour and Communication Survey	2002	12	Moderate
	2005	12	Moderate
	2008	12	Moderate
	2012	12	Moderate

A further source of bias in survey data relates to the use of *sampling weights*. All surveys considered in this study collected data using a multi-stage clustered sampling design and included stratification and oversampling of sub-populations of specific interest. Each dataset included, as per common practice, a set of sampling weights calculated in order to inflate the sample and represent the target population, taking into account not only the survey design (i.e. the inverse of the probability of being included in the sample, given the survey design) but also (as far as possible) non-response and coverage errors. To obtain the latter result, the weights provided with the surveys are *calibrated*, i.e. adjusted so that their sum across specific population strata (defined by sex, age category, race and province in our datasets) matched externally supplied population totals. Unfortunately, it is known that the population totals used for the calibration of weight in the various surveys do not represent a consistent temporal series, because the different surveys used the population totals available at that point in time from the national statistics agency (Statistics South Africa, http://www.statssa.gov.za) which are periodically adjusted when more precise data become available from a census or other sources.[26] This might introduce bias when, as in this case, the interest lies in the joint analysis of multiple datasets.

3 Additional methods

3.1 Recalibration of sampling weights

We used the population totals described in Section 2.3 to recalibrate the sampling weights in each survey in order to match as closely as possible the gender and 5-years age group distribution in each of the 9 Provinces (first level of administrative subdivision) of South Africa in the corresponding year. We used the Deville and Sarndal calibration method described by Pacifico[27] and implemented in the Stata^{R}[28] command *reweight* and we bound the resulting weights to the interval [0.2;-5].

The iterative calibration algorithm converged in all cases with a number of iterations between 25 and 345 and tolerance $< 10E\text{-}7$. Figure A1 illustrates the differences between the original and recalibrated population totals per sex and age group.

Figure A1: Original vs. recalibrated population totals.

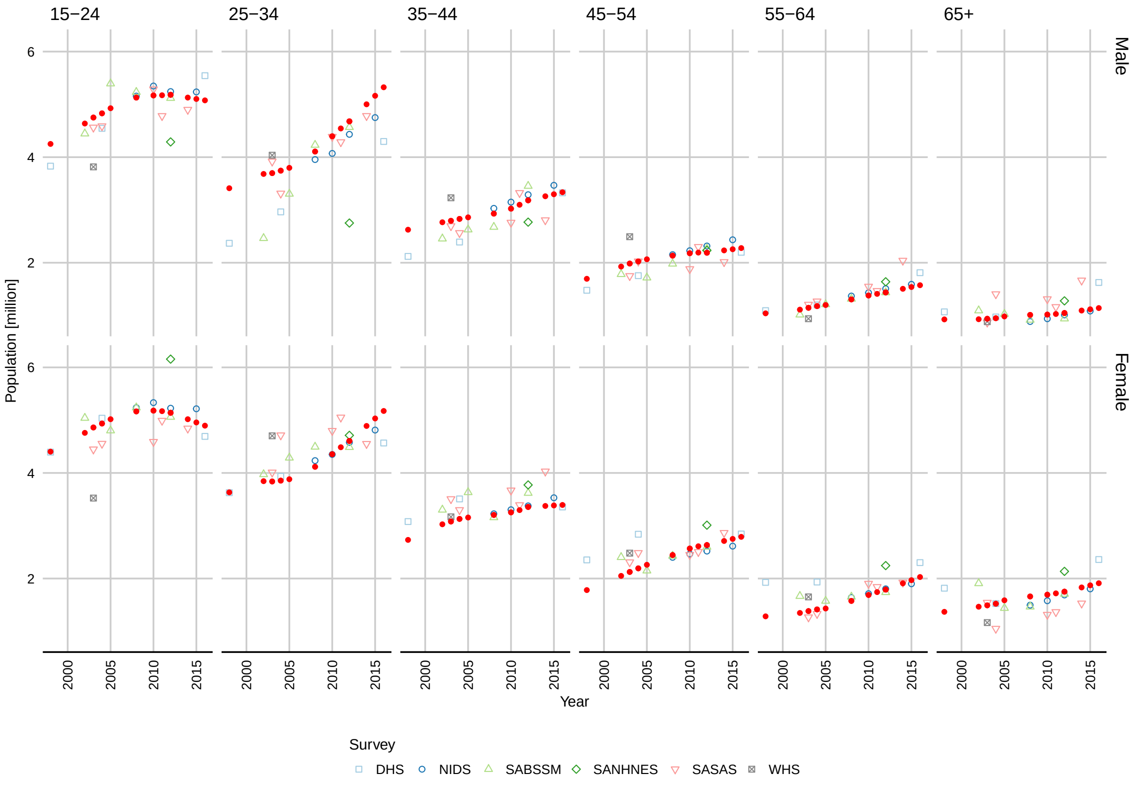

hollow marks = original; red dots: recalibrated.

3.2 Extracting drinking status form survey items

Table A6 summarises the strategy used to recover a binary indicator of 'current drinking' at individual level from the response to the relevant items in each survey dataset.

Table A6: Definition of 'current drinking status' in each survey

Survey	Items	Current Drinker?	
		Yes	No
WHS 2003	**q4010**: *Have you ever consumed a drink that contains alcohol such as beer, wine, etc.?* ; **q4011** – **q4017**: *How many standard drinks of any alcoholic beverage did you have on Monday, Tuesday, Wednesday, Thursday, Friday, Saturday, Sunday?*	q4011 != 0 or q4012 != 0 or q4013 != 0 or q4014 != 0 or q4015 != 0 or q4016 != 0 or q4017 != 0	(q4011 = 0 and q4012 = 0 and q4013 = 0 and q4014 = 0 and q4015 = 0 and q4016 = 0 and q4017 = 0) or q4010 = 'No'
SADHS 1998	**q086**: *Have you ever drunk alcohol?* ; **q087**: *Do you drink alcohol now?*	q087 = 'Yes'	q087 = 'No' or q086 = 'No'
SADHS 2003	**q42A**: *Have you ever consumed a drink that contains alcohol (such as beer, wine, spirits or sorghum beer)?* ; **q42B**: *Was this within the past 12 months?*	q42B = 'Yes'	q42B = 'No' or q42A = 'No'
SADHS 2006	**q1224**: *Have you ever consumed a drink that contains alcohol (such as beer, wine, spirits or sorghum beer)?* ; **q1225**: *Was this within the past 12 months?*	q1225= 'Yes'	q1225 = 'No' or q1224 = 'No'
NIDS 2008	**J31**: *How often do you drink alcohol?* (I have never drunk alcohol → 1, I no longer drink alcohol → 2, I drink very rarely → 3, Less than once a week → 4, On 1 or 2 days a week → 5, On 3 or 4 days a week → 6, On 5 or 6 days a week → 7, Every day → 8)	J32 >2	J32 = 1 or J32 = 2
NIDS 2010-11	J28: How often do you drink alcohol? (I have never drunk alcohol → 1, I no longer drink alcohol → 2, I drink very rarely → 3, Less than once a week → 4, On 1 or 2 days a week → 5, On 3 or 4 days a week → 6, On 5 or 6 days a week → 7, Every day → 8)	J28>2	J28 = 1 or J28 = 2
NIDS 2012	**J27**: *How often do you drink alcohol?* (I have never drunk alcohol → 1, I no longer drink alcohol → 2, I drink very rarely → 3, Less than once a week → 4, On 1 or 2 days a week → 5, On 3 or 4 days a week → 6, On 5 or 6 days a week → 7, Every day → 8)	J27 >2	J27 = 1 or J27 = 2
NIDS 2014-15	**J27**: *How often do you drink alcohol?* (I have never drunk alcohol → 1, I no longer drink alcohol → 2, I drink very rarely → 3, Less than once a week → 4, On 1 or 2 days a week → 5, On 3 or 4 days a week → 6, On 5 or 6 days a week → 7, Every day → 8)	J27 >2	J27 = 1 or J27 = 2
SASAS 2003	**q127**: *Do you do any of the following activities: Drink alcohol?* (Often = 1, Sometimes = 2, Never = 3)	q127< 3	q127 = 3

SASAS 2004	**q127**: *Do you do any of the following activities: Drink alcohol?* (Often = 1, Sometimes = 2, Never = 3)	q127< 3	q127 = 3
SASAS 2010	**q218**: *In the past 12 months, how frequently have you had at least one drink?* (5 or more days a week = 1, 1-4 days a week =2, 1-3 days a month = 3, Less than once a month = 4, Not currently consuming alcohol = 5, Never consumed alcohol = 8)	q218 < 5	q218 = 5 or q218 = 8
SASAS 2014	**q8**: *In the last 12 months, how often have you had a drink containing alcohol?* (Every day = 1, Several times a week = 2, Once a week = 3, 2-3 times a month = 4, Once a month = 5, Less than once a month = 6, Never = 7	q8 < 7	q8 = 7
SANHNES 2012	**B7.1**: *How often did you have a drink containing alcohol in the past 12 months?* (Never = 0, Monthly or less = 1, 2-4 times a month = 2, 2-3 times a week = 3, 4 or more times a week = 4)	B7.1 > 0	B7.1 = 0
SABSSM 2002	**q16.1**: *Do you drink alcohol*?	q16.1 = 'Yes'	q16.1 = 'No'
SABSSM 2005	**q11.1**: *Have you ever had a drink containing alcohol?* ; ***q11.2:*** How often did you have a drink containing alcohol in the past 12 months? (Not in the past 12 months = 1, Once a month or less = 2, 2-4 times a month = 3, 2-3 times a week = 4, 4 or more times a week = 5)	q11.2 > 1	q11.2 = 1 or q11.1 = 'No'
SABSSM 2008	**q10.1**: *Have you ever had a drink containing alcohol?* ; **q10.2**: *How often did you have a drink containing alcohol in the past 12 months?* (Not in the past 12 months = 1, Once a month or less = 2, 2-4 times a month = 3, 2-3 times a week = 4, 4 or more times a week = 5)	q10.2 > 1	q10.2 = 1 or q10.1 = 'No'
SABSSM 2012	**q12.1**: *Have you ever had a drink containing alcohol?* **q11.2**: *How often did you have a drink containing alcohol in the past 12 months?* (Not in the past 12 months = 1, Once a month or less = 2, 2-4 times a month = 3, 2-3 times a week = 4, 4 or more times a week = 5)	q11.2 > 1	q11.2 = 1 or q11.1 = 'No'

3.3 Calculating consumption intervals

For surveys that collected data with frequency/quantity questionnaires, we calculate individual *consumption intervals* by multiplying the bounds of the *frequency interval* by the bounds of the *typical quantity* interval and converting the result in g/day.

For example, if an individual indicated that he/she drunk between 2 and 3 times a week, and the typical quantity consumed in a drinking occasion was between 1 and 2 standard drinks, the resulting consumption interval ranged between:

$$2\,\frac{occasion}{week}\cdot 1\,\frac{standard\,drink}{occasion}\cdot 12\,\frac{g}{standard\,drink} = 24\,\frac{g}{week} = 3.4\,\frac{g}{day} \qquad \text{[lower bound]}$$

and

$$3\,\frac{occasion}{week}\cdot 2\,\frac{standard\,drink}{occasion}\cdot 12\,\frac{g}{standard\,drink} = 72\,\frac{g}{week} = 10.3\,\frac{g}{day} \qquad \text{[upper bound]}$$

3.4 Quality effect weighting and effective sample size

For the preprocessed datasets including the estimated proportion of individuals in each consumption class, we calculated quality effect weights with the procedure described by Doi et al.[29] We used the variance of the estimated proportion in each class as a measure of random variability (the ν_j in Doi's formulae) and the risk of bias scores from Table A5 rescaled to the 0-1 interval as a measure of the variability due to study bias (the Q_j in Doi's formulae).

We then calculated the effective sample size of each estimate (the $ne_{s,g,a}$ in the expression of the model likelihood function) by redistributing the total sample size across all surveys proportionally to the quality effect weights (the $w_j^{"}$ in Doi's formulae).

3.5 Parametrization of the GAM and choice of the number of bases

In both the consumption and prevalence splines, we impose relatively low 'flexibility' (i.e. less degrees of freedom) along the temporal axis, deeming implausible large variations year-by-year. Conversely, we allowed higher flexibility (more degrees of freedom) on the age axis, because of the evidence from various populations that relatively sharp variations in alcohol consumption patterns with age are common, especially at younger ages.[30] This translated in the following choices for the number of bases:

$$dc_1 = 3\ ;\ dc_2 = 5\ ;\ dp_1 = 3\ ;\ dp_2 = 5 \tag{A3}$$

As a sensitivity analysis, we re-fitted the model with increased dimension for the time bases ($dc_1 = dp_1 = 5$), and the results shown in Section 4.2.1 did not alter the substantive conclusions regarding time and age patterns in the population.

We implemented the GAMs described in Eq. 7 and 8 in the main text using the mixed effects parametrization from the R package *mgcv*.[31] In this representation, the wiggly parts of the spline basis are treated as a random effect and their associated variance parameter controls the degree of wiggliness of the fitted spline. The perfectly smooth parts of the basis are treated as a fixed effect. With this parametrization, Eq. 7 becomes (vectors and matrices in **bold**):

$$\boldsymbol{\mu_i} = exp(b_i + \boldsymbol{Xs_i bs_i} + \boldsymbol{Zs_{g,1}} \cdot \boldsymbol{s_{g,1}} + \boldsymbol{Zs_{g,2}} \cdot \boldsymbol{s_{g,2}} + \boldsymbol{Zs_{g,3}} \cdot \boldsymbol{s_{g,3}}) \tag{A4}$$

where

b_g	is the intercept;
$\boldsymbol{bs_g}$	is a vector of coefficients (fixed effects) for the perfectly smooth part of the spline;
$\boldsymbol{s_{g,1}}, \boldsymbol{s_{g,2}}, \boldsymbol{s_{g,3}}$	are vectors of coefficients for the wiggly part of the spline (random effects) modelled as the product of a vector of standardised coefficients $\boldsymbol{zs_{g,\cdot}}$ times their standard deviation $sds_{g,\cdot}$: $Zsds_{g,\cdot} = sds_{g,\cdot} \cdot \boldsymbol{zs_{g,\cdot}}$;
$\boldsymbol{Xs_g}$, $\boldsymbol{Zs_{g,1}}$, $\boldsymbol{Zs_{g,2}}$, $\boldsymbol{Zs_{g,3}}$	are the design matrices of the thin plate splines;
$\boldsymbol{\mu_g}$	is the mean of the consumption distribution;
$g \in \{1, 2\}$	is the sex indicator.

With the choice of number of bases indicated above, the model A4 has a total of 15 coefficients to be estimated.

The model in Eq. 8 of the Article is parametrized in the same way.

3.6 Soft-capping the distribution

To formally translate the assumption that average consumptions of more than 150 g/day of pure alcohol are extremely unlikely, we impose the prior shown is Figure A2 to the 97.5th percentile of the population distribution.

Figure A2: Custom prior distribution for the 97.5th percentile of the distributions of average alcohol consumption among drinkers.

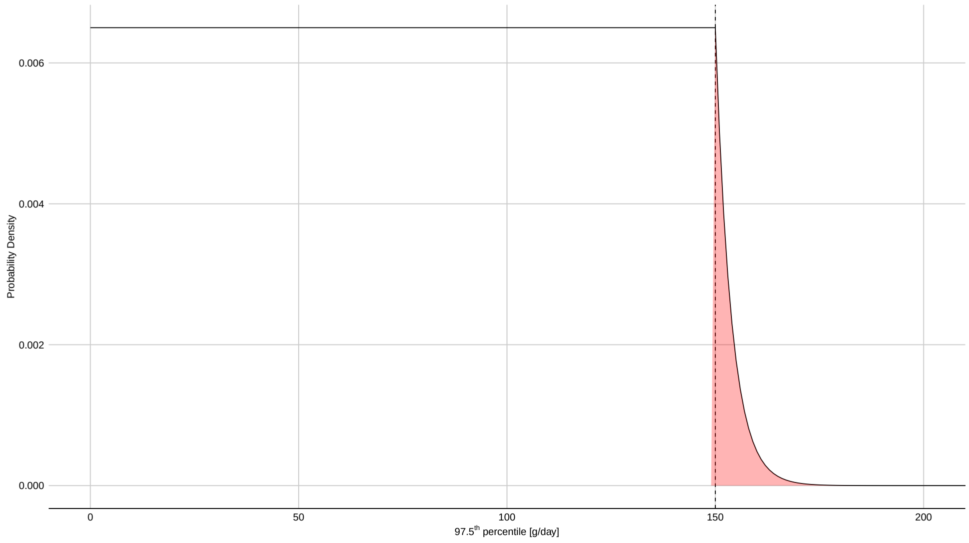

The prior is uniformly distributed in the interval (0; 150] g/day with 97.5% of probability and

decays exponentially for higher values. It is formally defined by Equation A5.

$$f(x) = \begin{cases} \dfrac{0.975}{150} & x < 150 \\ \\ 0.0250e^{-0.26(x-150)} & x >= 150 \end{cases} \tag{A5}$$

Because no closed form exists that expresses the quantiles of the Gamma distribution as a function of its mean μ and shape α, imposing exactly this constraint would have required solving numerically an equation at each iteration during the sampling process. To reduce the computational burden, and given the limited range of μ and α of interest in our study, we considered the following linear function to approximate the relationship in that range:

$$97.5^{th}\ percentile = \beta_0 + \beta_1\mu + \beta_2\alpha + \beta_3\mu\alpha \tag{A6}$$

and we recovered the best-fitting parameters (for $\alpha \in [0.5; 0.9]$ and $\mu \in [10; 350]$) with ordinary least square:

$$\begin{cases} \beta_0 = 3.259e^{-12} \\ \beta_0 = 6.397 \\ \beta_0 = -1.887e^{-12} \\ \beta_0 = -2.884 \end{cases} \tag{A7}$$

We deemed the result (see Figure A3) adequate for the purpose.

Figure A3: Approximate linear relationship between mean and 97.5th percentile of the distribution of average alcohol consumption among drinkers for different values of the shape parameter α.

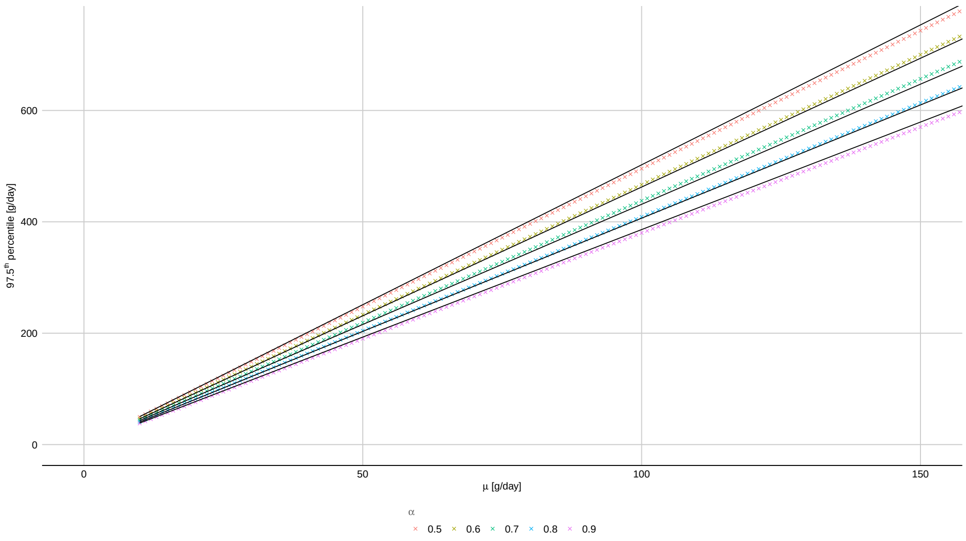

Solid lines: exact percentile (by numerical solution of the equation); + = model-predicted percentile

3.7 Priors

Table A7 lists the prior distributions assigned to the 324 model parameters.

Table A7: Prior distribution of model parameters.

Parameter	Type	Dim	Description	Prior	Notes
$alpha_1$	matrix	19*6	Shape parameter of the Gamma distribution of alcohol consumption among drinkers, per year and age group (males).	$\mathcal{N}(0.73, 0.028)$	Informative prior. It reflects the estimates by Kehoe et Al. of the distribution of the ratio $sd//mu$ in the distribution of alcohol consumption across populations. The standard deviation is a first order approximation of the standard deviation of the inverse of that ratio.
$alpha_2$	matrix	19*6	Shape parameter of the Gamma distribution of alcohol consumption among drinkers, per year and age group (females).	$\mathcal{N}(0.63, 0.036)$	
cov	vector	12	Coverage, per survey.	$\mathcal{N}(0.5, 0.5)$	Weakly informative prior.
$covdev$	vector	12	Coverage deviation, per sex and age.	$\mathcal{N}(1, 0.02)$	Informative prior. Expresses the hypothesis that the coverage varies only modestly across groups, by making deviations $> \pm 5\%$ very unlikely.
b	vector	2	Intercepts of the linear predictor of the mean consumption among drinkers (males, females).	$\mathcal{N}(3, 3)$	Weakly informative prior. Centered at 3 in the log scale (≈ 20 in the natural scale), with large variance.
bs	vector	6	Coefficients (fixed effects) of the smooth part of the thin plate spline for the mean consumption among drinkers (males, females).	$t(3, 0, 1)$	Weakly informative prior.
$zs_{1,1}$<br>$zs_{1,2}$<br>$zs_{1,3}$<br>$zs_{2,1}$<br>$zs_{2,2}$<br>$zs_{2,3}$	vector<br>vector<br>vector<br>vector<br>vector<br>vector	3<br>6<br>2<br>3<br>6<br>2	Standardised coefficients (random effects) for the wiggly part of the thin plate spline for the mean consumption among drinkers (males, females).	$\mathcal{N}(0, 1)$	Weakly informative prior.
$sds_{1,1}$<br>$sds_{1,2}$<br>$sds_{1,3}$<br>$sds_{2,1}$<br>$sds_{2,2}$<br>$sds_{2,3}$	scalar<br>scalar<br>scalar<br>scalar<br>scalar<br>scalar	1<br>1<br>1<br>1<br>1<br>1	Standard deviation of the random effect of the thin plate spline for the mean consumption among drinkers (males, females).	$t(3, 0, 1)T[0, +\inf]$	Weakly informative prior.
bp	vector	2	Intercepts of the linear predictor of the prevalence of drinkers (males, females).	$\mathcal{N}(0, 0.5)$	Weakly informative prior. Centered at 0 in the logit scale.
bsp	vector	6	Coefficients (fixed effects) of the smooth part of the thin plate spline for the prevalence of drinkers (males, females).	$t(3, 0, 1)$	Weakly informative prior.
$zsp_{1,1}$<br>$zsp_{1,2}$<br>$zsp_{1,3}$<br>$zsp_{2,1}$<br>$zsp_{2,2}$<br>$zsp_{2,3}$	vector<br>vector<br>vector<br>vector<br>vector<br>vector	3<br>6<br>2<br>3<br>6<br>6	Standardised coefficients (random effects) for the wiggly part of the thin plate spline for the prevalence of drinkers (males, females).	$\mathcal{N}(0, 1)$	Weakly informative prior.
$sdsp_{1,1}$<br>$sdsp_{1,2}$<br>$sdsp_{1,3}$<br>$sdsp_{2,1}$<br>$sdsp_{2,2}$<br>$sdsp_{2,3}$	scalar<br>scalar<br>scalar<br>scalar<br>scalar<br>scalar	1<br>1<br>1<br>1<br>1<br>1	Standard deviation of the random effect of the thin plate spline for the prevalence of drinkers (males, females).	$\mathcal{N}(0, 1)T[0, +\inf]$	Weakly informative prior.

3.8 Stan model and R code

The Stan model and the associated R code used to produce the results presented in this article are available on request from the corresponding author (acois@sun.ac.za).

4 Additional results

4.1 Model Checking

4.1.1 Convergence statistics and posterior distribution of parameters

Figure A4 shows the trace plot for the log-likelihood, its posterior distribution and the autocorrelation between draws.

Figure A4: Posterior log-likelihood*: trace , density, autocorrelation plot.

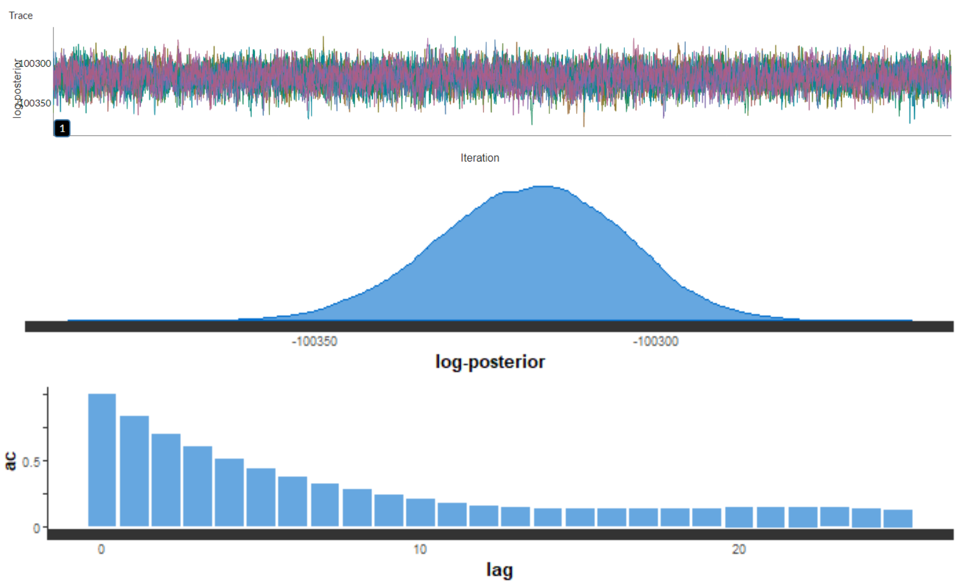

* Un-normalised. Plots exclude warm-up samples.

The potential scale reduction statistic $\hat{R}$ varied between 1.000 and 1.023 across the 856 parameters and the effective sample size ECC between 539.7 and 13613.7. Dataset 4 in Additional File 2 shows summary statistics for the distribution of each parameter.

4.1.2 Prevalence/consumption estimates: residuals and posterior predictive check

Figure A5 shows the quantile-quantile plots for the residuals of the prevalence estimates from each survey. Figure A6 shows the QQ plots for the *randomised* quantile residuals of the consumption estimates.[32] Randomised residuals are reported in the latter case because the interval-censored nature of the input data makes the calculation of ordinary probability residuals not meaningful (see: https://mjskay.github.io/tidybayes/articles/tidybayes-residuals.html

[accessed: 27/10/2020]).

Figure A5: Standardised residuals for the prevalence of drinkers: QQ plots. Per survey.

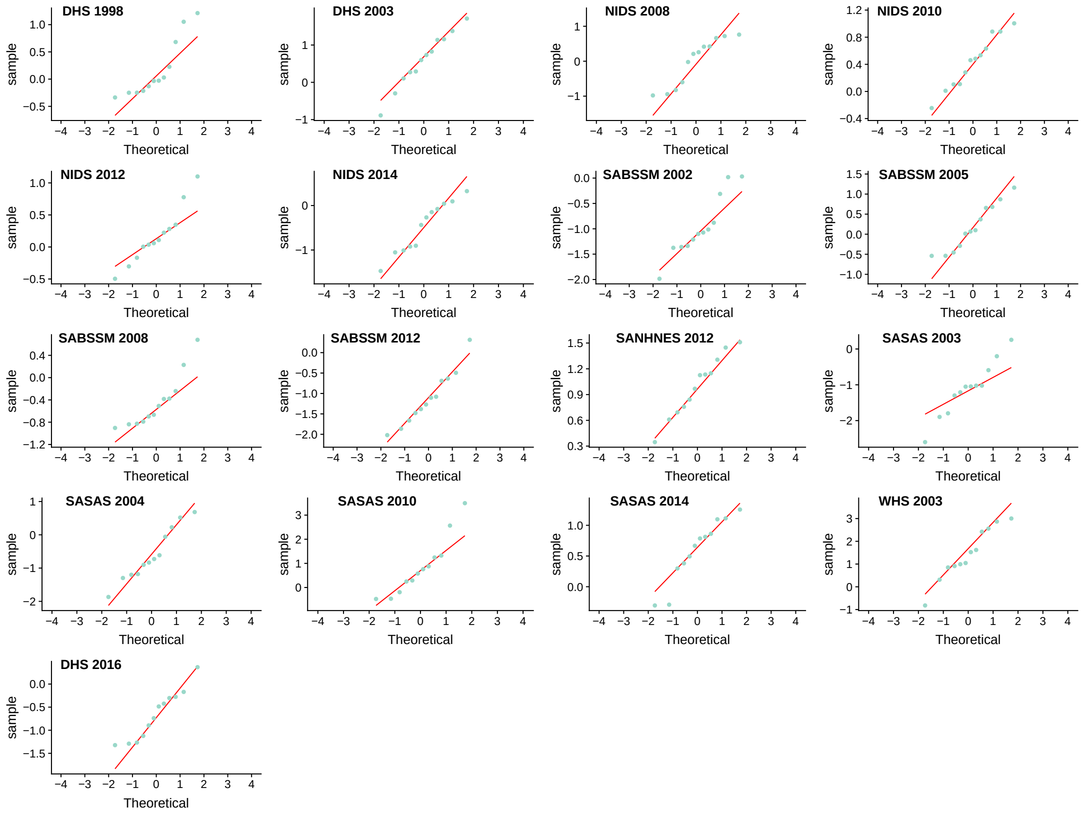

Figure A6: Randomised Quantile Residual for average consumption by drinkers: QQ plots. Per survey.

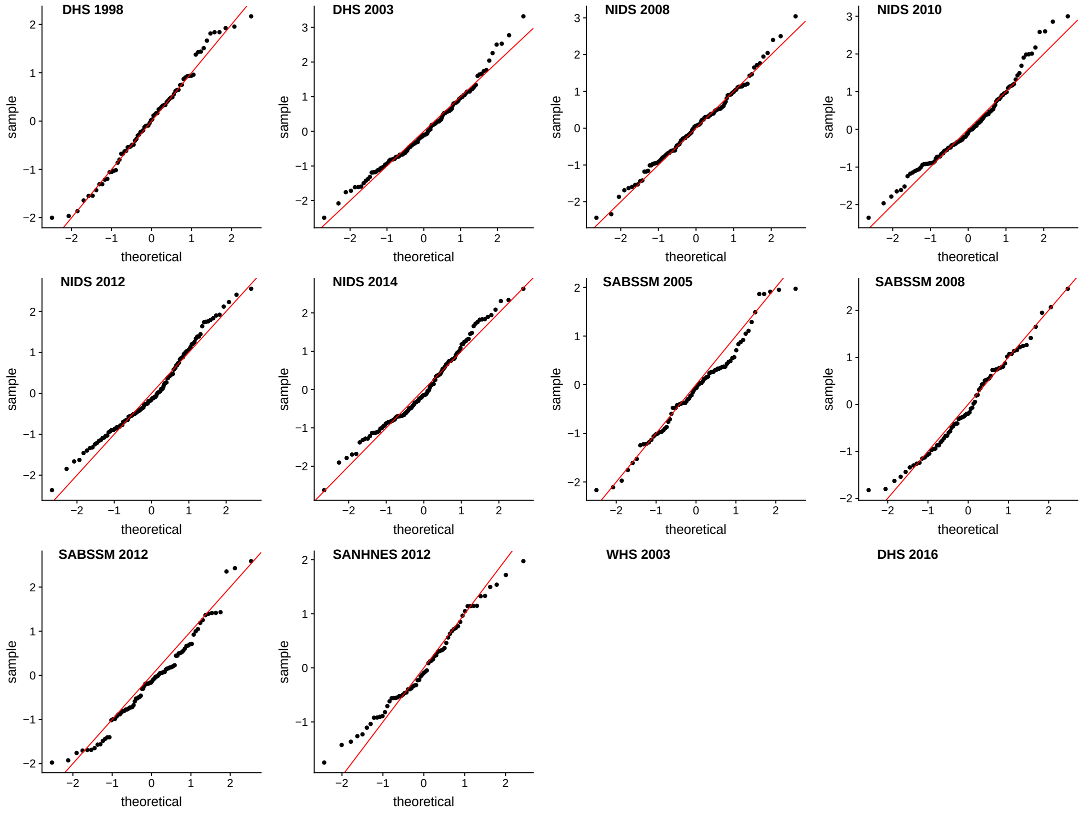

Figure A7 compares the observed cumulative distribution of average consumption of alcohol among drinkers from each survey (recovered non-parametrically for the surveys with censored consumption data) with the distribution predicted by the model.

Figure A7: Posterior Predictive Check: Observed vs. predicted cumulative distribution of average alcohol consumption among drinkers. Per survey.

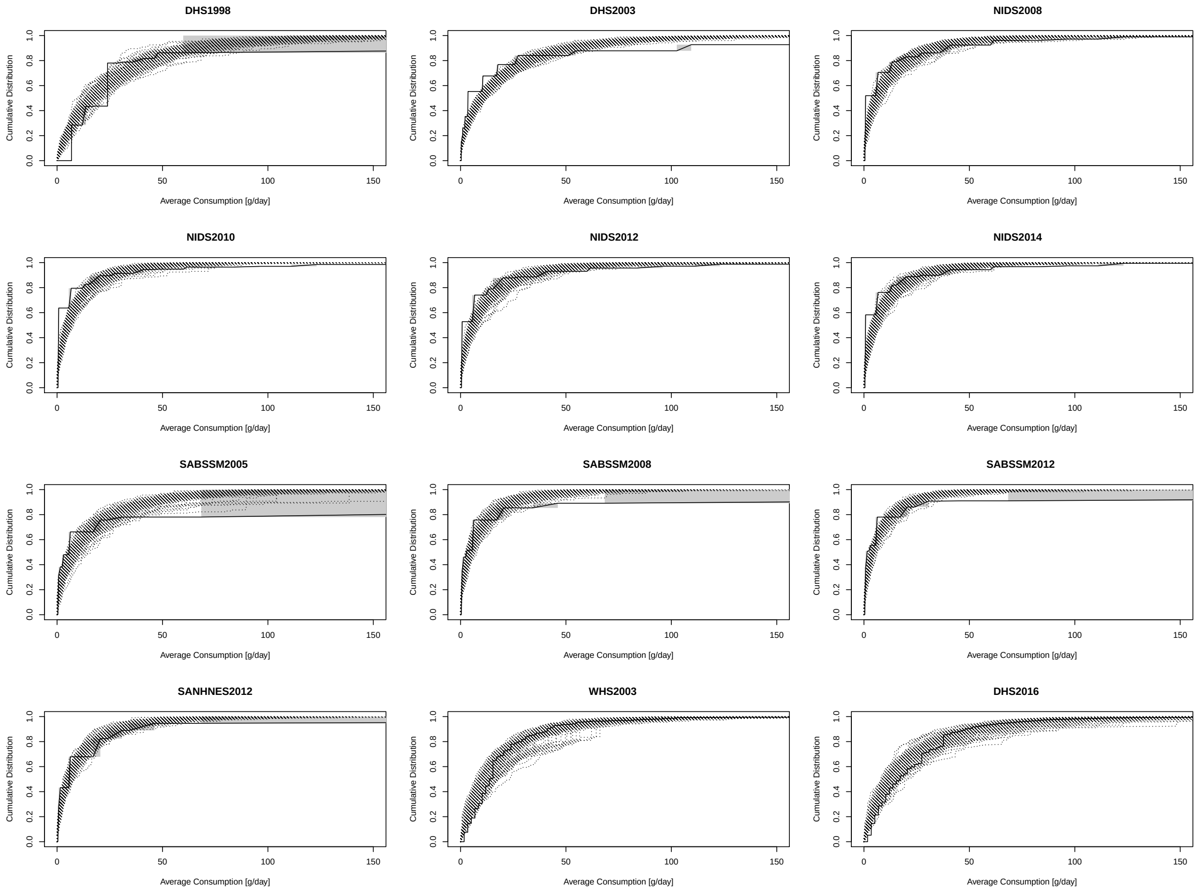

Solid line: observed distribution; Dotted lines: 100 random draws from the posterior distribution. The gray areas represent the zones of uncertainty in the observed distributions due to censoring.

4.2 Sensitivity analysis

4.2.1 Increasing spline bases dimension

Figure A8 shows the predicted trends in prevalence of drinking and average consumption among drinkers when the dimension of the spline bases is increased. In both cases the results are substantively similar to the results of the main analysis.

Figure A8: Trends in prevalence of drinkers. South Africa, population 15+, 1998-2016. Per sex and age category.
Sensitivity analysis for increased spline base dimension ($dc_1 = 5$; $dc_2 = 5$; $dp_1 = 5$; $dp_2 = 5$)

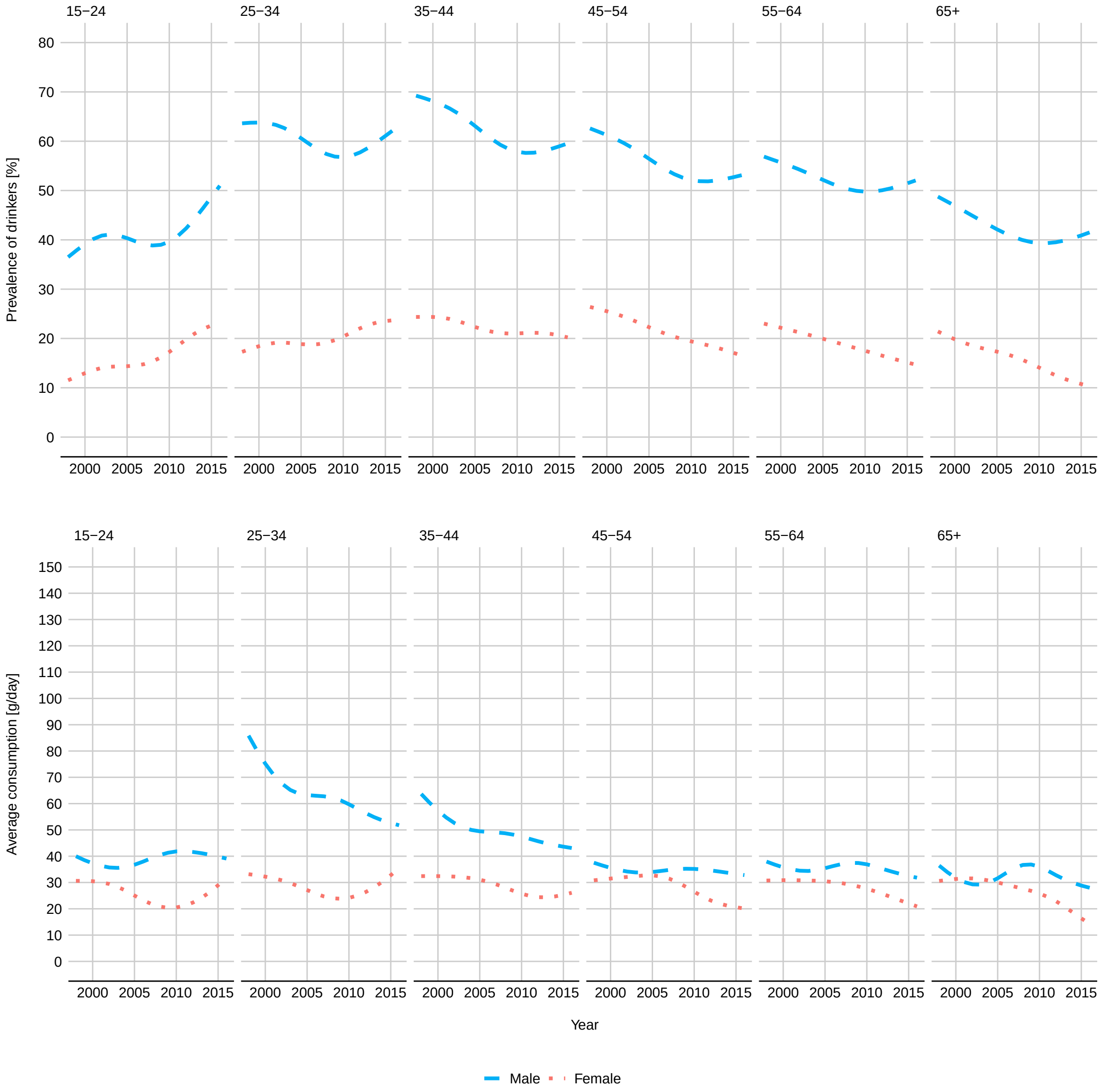

4.2.2 Prevalence and consumption assuming no wasted alcohol

Figure A9 shows how trends estimates changed when we dropped the assumption of a 20% wastage of alcohol, and we assumed than all APC was actually used for drinking.

Figure A9: Trends in prevalence of drinkers and average consumption among drinkers. South Africa, population 15+, 1998-2016. Per sex and age category.
Sensitivity analysis: assumption of no wasted alcohol.

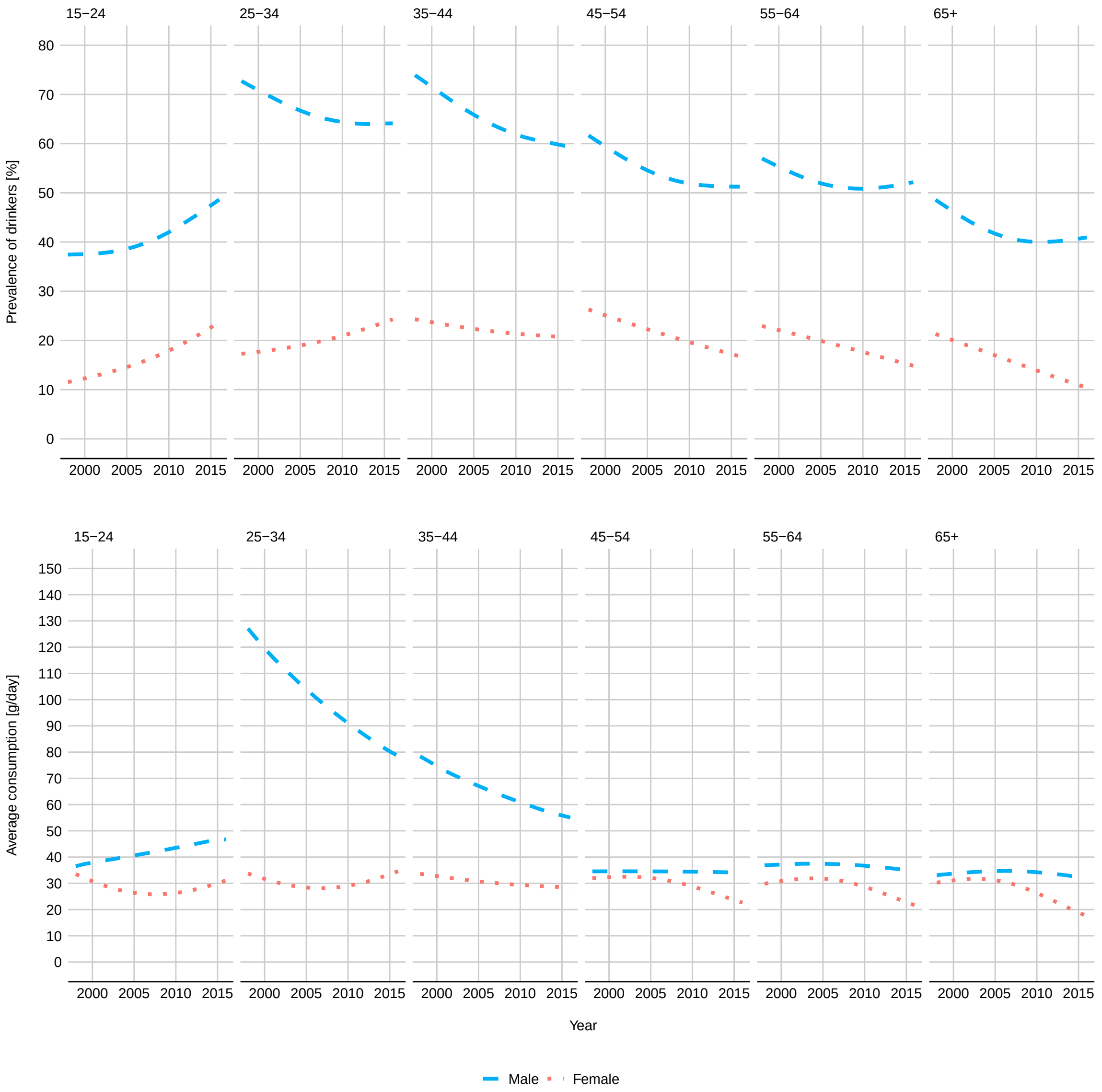

The new estimates show limited (if any) change in the prevalence across all age classes, and, as expected, a marked overall increase in the average consumption. Interestingly, the increase is not distributed uniformly across age categories and sexes. On the contrary, it appears mostly concentrated in the highest consumption categories, i.e. men 25-44 years.

Table A8 shows the differences in prevalence of drinkers and average consumption among drinkers for selected years estimated by the model in the the two hypotheses of 20% wastage and no wastage.

Table A8: Differences in the estimated prevalence of drinkers and average alcohol consumption among drinkers in the hypothesis of 20% vs. 0% wastage. Year 1998, 2008,2016. By age and sex.

		Prevalence			Consumption		
Year	Age	20%	0%	Difference [%]	20%	0%	Difference [g/day]
**Males:**							
1998	15-24	37.8	37.4	-0.4	37.2	36.5	-0.7
1998	25-34	66.0	72.7	+6.7	82.2	127.1	+44.9
1998	35-44	70.7	73.9	+3.3	60.8	78.4	+17.6
1998	45-54	63.1	61.7	-1.5	35.7	34.5	-1.2
1998	55-64	57.6	57.0	-0.6	36.3	36.9	+0.
1998	65+	49.0	48.6	-0.5	33.3	33.1	-0.2
2008	15-24	39.3	40.3	+1.0	37.1	42.3	+5.2
2008	25-34	59.2	65.1	+5.8	60.7	95.9	+35.3
2008	35-44	60.0	63.2	+3.3	48.5	63.3	+14.73
2008	45-54	53.0	52.6	-0.3	34.4	34.5	+0.1
2008	55-64	50.5	51.0	+0.5	35.7	37.1	+1.5
2008	65+	40.1	40.3	+0.2	33.8	34.6	+0.8
2016	15-24	48.3	48.6	+0.3	41.6	46.7	+5.1
2016	25-34	61.0	64.1	+3.2	52.7	78.5	+25.8
2016	35-44	57.9	59.5	+1.6	43.6	55.1	+11.4
2016	45-54	51.7	51.2	-0.4	33.8	34.1	+0.3
2016	55-64	51.9	52.2	+0.2	33.2	34.7	+1.5
2016	65+	40.8	40.9	+0.1	30.6	32.0	+1.5
**Females:**							
1998	15-24	11.7	11.5	-0.1	33.2	33.5	+0.3
1998	25-34	17.5	17.3	-0.2	34.1	33.7	-0.4
1998	35-44	24.6	24.3	-0.3	33.8	33.6	-0.2
1998	45-54	26.5	26.3	-0.2	31.6	32	+0.4
1998	55-64	23.0	22.9	-0.1	29.4	29.8	+0.5
1998	65+	21.5	21.3	-0.2	30.4	30.3	-0.1
2008	15-24	16.2	16.4	+0.2	23.4	25.8	+2.4
2008	25-34	20.0	20.1	+0.1	26.8	28.3	+1.5
2008	35-44	21.7	21.7	+0	28.9	29.8	+1.0
2008	45-54	20.6	20.7	+0.1	29.5	30.5	+1.0
2008	55-64	18.5	18.6	+0.1	29.4	30.4	+1.0
2008	65+	15.2	15.2	-0.0	27.8	28.8	+1.1
2016	15-24	23.4	23.7	+0.3	29.4	31.1	1.7
2016	25-34	24.0	24.3	+0.3	33.5	34.6	1.1
2016	35-44	20.4	20.6	+0.2	26.3	28.4	2.2
2016	45-54	16.6	16.8	+0.2	20.2	22.6	2.4
2016	55-64	14.7	14.9	+0.1	19.8	21.6	1.9
2016	65+	10.2	10.4	+0.2	15.7	17.5	1.8

4.3 Consumption by drinking category

Table A9 shows the average alcohol consumption by light, intermediate and heavy drinkers, and the proportion of the total consumption at country level accounted for by each category.

Table A9: Average alcohol consumption and proportion of total volume consumed at country level per drinking category. By year and sex.

	Average consumption [g/day]			Proportion of total [%]		
Year	Light	Inter	Heavy	Light	Inter	Heavy
**Males:**						
1998	9.9	39.6	72.9	16.6	10.3	73.2
1999	9.5	39.6	72.6	16.8	9.8	73.4
2000	9.5	39.6	72.9	17.0	9.8	73.2
2001	9.5	39.5	73.3	17.1	9.9	73.0
2002	9.5	39.5	73.6	17.3	10.0	72.7
2003	10.0	39.5	75.3	17.3	10.9	71.8
2004	8.9	39.5	72.9	17.8	9.2	72.9
2005	8.6	39.4	72.4	18.1	8.9	73.0
2006	9.4	39.4	74.8	17.9	10.2	71.9
2007	9.4	39.4	75.0	18.0	10.2	71.8
2008	8.2	39.4	71.8	18.4	8.4	73.1
2009	9.4	39.4	75.3	18.1	10.2	71.6
2010	8.6	39.4	73.3	18.4	9.1	72.5
2011	9.4	39.4	75.5	18.2	10.3	71.6
2012	8.2	39.4	72.3	18.5	8.5	73.0
2013	9.4	39.4	75.6	18.2	10.3	71.6
2014	9.4	39.4	75.7	18.2	10.3	71.5
2015	8.7	39.4	73.8	18.5	9.2	72.4
2016	9.8	39.4	76.7	18.3	11.0	70.7
**Females:**						
1998	14.4	30.9	46.7	21.8	21.4	56.7
1999	12.0	30.5	47.9	22.4	17.6	60.0
2000	11.8	30.2	47.6	23.0	17.7	59.3
2001	11.7	29.9	47.2	23.7	17.9	58.4
2002	11.7	29.6	46.8	24.3	18.2	57.5
2003	14.3	29.3	43.8	25.3	24.1	50.6
2004	9.5	29.3	47.8	24.8	14.3	60.8
2005	9.0	29.1	47.4	25.2	13.8	61.0
2006	11.4	28.7	45.5	26.5	19.0	54.5
2007	11.4	28.6	45.3	26.9	19.2	54.0
2008	9.0	28.7	47.3	26.2	14.3	59.5
2009	11.3	28.3	45.0	27.4	19.4	53.2
2010	9.3	28.6	47.0	26.7	14.9	58.5
2011	11.3	28.2	44.7	27.8	19.6	52.6
2012	9.1	28.5	47.0	26.9	14.6	58.5
2013	11.3	28.1	44.4	28.2	19.7	52.1
2014	11.3	28.0	44.2	28.6	19.7	51.7
2015	9.3	28.3	46.1	27.9	15.3	56.8
2016	13.2	27.5	41.2	30.0	24.2	45.8

Light = *Light drinkers*: average daily consumption $< 12/24$ g; Inter = *Intermediate drinkers*: average daily consumption $\geq 12/24$ g and $< 40/60$ g; Heavy *Heavy drinkers*: average daily consumption $> 40/60$ g. The first figure refers to females, the second to males.

5 References

[1] Stevens GA, Alkema L, Black RE, Boerma JT, Collins GS, Ezzati M, et al. Guidelines for Accurate and Transparent Health Estimates Reporting: the GATHER statement. The Lancet. 2016;388(10062):e19–e23. Available from: https://doi.org/10.1016/S0140-6736(16)30388-9.

[2] WHO Multi-Country Studies Data Archive. South Africa World Health Survey 2003, Wave 0 [Dataset];. Available from: https://apps.who.int/healthinfo/systems/surveydata/index.php/catalog/71.

[3] Department of Health. South Africa Demographic and Health Survey 1998 [Dataset];. Available from: https://dhsprogram.com/methodology/survey/survey-display-113.cfm.

[4] National Department of Health, South African Medical Research Council, OrcMacro. South Africa Demographic and Health Survey 2003.;. Available from: https://dhsprogram.com/pubs/pdf/FR206/FR206.pdfhttps://www.cambridge.org/core/product/identifier/CBO9781107415324A009/type/book_part.

[5] National Department of Health , Statistics South Africa, South African Medical Research Council and ICF. South Africa Demographic and Health Survey 2016: Report. National Department of Health; 2019.

[6] Southern Africa Labour and Development Research Unit. National Income Dynamics Study (NIDS) Wave 1, 2008. Version 7.0.0. [Dataset];. Available from: https://doi.org/10.25828/e7w9-m033.

[7] Southern Africa Labour and Development Research Unit. National Income Dynamics Study Wave 2, 2010-2011. Version 4.0.0. [Dataset];. Available from: https://doi.org/10.25828/j1h1-5m16.

[8] Southern Africa Labour and Development Research Unit. National Income Dynamics Study Wave 3, 2012. Version 3.0.0. [Dataset];. Available from: https://doi.org/10.25828/7pgq-q106.

[9] Southern Africa Labour and Development Research Unit. National Income Dynamics Study 2014-2015, Wave 4. Version 2.0.0. [Dataset];. Available from: https://doi.org/10.25828/f4ws-8a78.

[10] Human Sciences Research Council. South African Social Attitudes Survey (SASAS) 2003 [Dataset];. Available from: http://datacuration.hsrc.ac.za/.

[11] Human Sciences Research Council. South African Social Attitudes Survey (SASAS) 2004 [Dataset];. Available from: http://datacuration.hsrc.ac.za/.

[12] Human Sciences Research Council. South African Social Attitudes Survey (SASAS) 2010 [Dataset];. Available from: http://datacuration.hsrc.ac.za/.

[13] Human Sciences Research Council. South African Social Attitudes Survey (SASAS) 2014[Dataset];. Available from: http://datacuration.hsrc.ac.za/.

[14] Human Sciences Research Council. The South African National Health and Nutrition Examination Survey (SANHANES-1) [Dataset];. Available from: http://datacuration.hsrc.ac.za/.

[15] Human Sciences Research Council. South African HIV/AIDS, Behavioural Risks, Sero-status, and Mass Media Impact Survey (SABSSM) 2002: Guardian data - All provinces [Dataset];. Available from: http://datacuration.hsrc.ac.za/.

[16] Human Sciences Research Council. South African national HIV prevalence, HIV incidence, behaviour and communication survey, 2005 [Dataset];. Available from: http://datacuration.hsrc.ac.za/.

[17] Human Sciences Research Council. South African national HIV prevalence, incidence, behaviour and communication survey, 2008 [Dataset];. Available from: http://datacuration.hsrc.ac.za/.

[18] Human Sciences Research Council. South African National HIV Prevalence, Incidence and Behaviour Survey, 2012 [Dataset];. Available from: http://datacuration.hsrc.ac.za/.

[19] Van Heerden MS, Grimsrud AT, Seedat S, Myer L, Williams DR, Stein DJ. Patterns of Substance Use in South Africa: Results from the South African Stress and Health Study. South African Medical Journal. 2009;99(5 Pt 2):358–366.

[20] Manthey J, Shield KD, Rylett M, Hasan OSM, Probst C, Rehm J. Global alcohol exposure between 1990 and 2017 and forecasts until 2030: a modelling study. The Lancet. 2019;393(10190):2493–2502. Available from: https://doi.org/10.1016/S0140-6736(18)32744-2.

[21] World Health Organization. Global status report on alcohol and health 2018. World Health Organization; 2018. Available from: https://apps.who.int/iris/bitstream/handle/10665/274603/9789241565639-eng.pdf?ua=1.

[22] Probst C, Shuper PA, Rehm J. Coverage of Alcohol Consumption by National Surveys in South Africa. Addiction. 2017;112(4):705–710.

[23] Ekholm O. Influence of the recall period on self-reported alcohol intake. European Journal of Clinical Nutrition. 2004;58(1):60–63. Available from: https://doi.org/10.1038/sj.ejcn.1601746.

[24] Stockwell T, Donath S, Cooper-Stanbury M, Chikritzhs T, Catalano P, Mateo C. Under-reporting of alcohol consumption in household surveys: a comparison of quantity-frequency, graduated-frequency and recent recall. Addiction. 2004 Aug;99(8):1024–1033. Available from: https://doi.org/10.1111/j.1360-0443.2004.00815.x.

[25] Pillay-van Wyk V, Roomaney RA, Awotiwon OF, Nglazi MD, Turawa E, Ebrahim AH, et al. Burden of Disease Review Manager for Systematic Review of Observational Studies: Technical Report and User Guide. Version 2. Cape Town: Burden of Disease Research Unit, South African Medical Research Council; 2018.

[26] Branson N, Wittenberg M. Re-Weighting South African National Household Survey Data to Create a Consistent Series over Time: A Cross Entropy Estimation Approach. Datafirst Technical Paper No. 15. Cape Town: Southern Africa Labour and Development Research Unit; 2011.

[27] Pacifico D. Reweight: a stata module to reweight survey data to external totals. Ministry of Economy and Finance; 2014. Available from: http://www.dt.tesoro.it/export/sites/sitodt/modules/documenti_it/analisi_progammazione/working_papers/WP_N._5_2014.pdf.

[28] StataCorp. Stata Statistical Software: Release 14. College Station, TX: StataCorp LP; 2014.

[29] Doi SAR, Barendregt JJ, Khan S, Thalib L, Williams GM. Advances in the Meta-Analysis of Heterogeneous Clinical Trials II: The Quality Effects Model. Contemporary clinical trials. 2015;45:123–129.

[30] Britton A, Ben-Shlomo Y, Benzeval M, Kuh D, Bell S. Life Course Trajectories of Alcohol Consumption in the United Kingdom Using Longitudinal Data from Nine Cohort Studies. BMC Med. 2015;13(1):47. Available from: https://doi.org/10.1186/s12916-015-0273-z.

[31] Wood SN. Generalized Additive Models: An Introduction with R. 2nd ed. Chapman and Hall/CRC; 2017.

[32] Dunn PK, Smyth GK. Randomized quantile residuals. Journal of Computational and Graphical Statistics. 1996;5(3):236–244.
